# Unit costs for non-acute care in Ireland 2016—2019

**DOI:** 10.12688/hrbopenres.13256.1

**Published:** 2021-04-23

**Authors:** Samantha Smith, Jingjing Jiang, Charles Normand, Ciaran O’Neill

**Affiliations:** 1 Centre for Health Policy and Management, Trinity College Dublin, Dublin, Ireland; 2 Cicely Saunders Institute, London, SE5 9PJ, UK; 3 Centre for Public Health, Queen’s University Belfast, Belfast, BT12 6BA, Ireland

**Keywords:** unit costs, micro-cost, bottom-up, non-acute, community, Ireland

## Abstract

**Background:** This paper presents detailed unit costs for 16 healthcare professionals in community-based non-acute services in Ireland for the years 2016—2019. Unit costs are important data inputs for assessments of health service performance and value for money. Internationally, while some countries have an established database of unit costs for healthcare, there is need for a more coordinated approach to calculating healthcare unit costs. In Ireland, detailed cost analysis of acute care is undertaken by the Healthcare Pricing Office but to date there has been no central database of unit costs for community-based non-acute healthcare services.

**Methods:** Unit costs for publicly employed allied healthcare professionals, Public Health Nurses and Health Care Assistant staff are calculated using a bottom-up micro-costing approach, drawing on methods outlined by the Personal Social Services Research Unit in the UK, and on available Irish and international costing guidelines. Data on salaries, working hours and other parameters are drawn from secondary datasets available from Department of Health, Health Service Executive and other public sources. Unit costs for public and private General Practitioner, dental, and long-term residential care (LTRC) are estimated drawing on available administrative and survey data.

**Results: **The unit costs for the publicly employed non-acute healthcare professionals have changed by 2–6% over the timeframe 2016–2019 while larger percentage changes are observed in the unit costs for public GP visits and public LTRC (14-15%).

**Conclusions:** The costs presented here are a first step towards establishing a central database of unit costs for non-acute healthcare services in Ireland. The database will help ensure consistency across Irish health costing studies and facilitate cross-study and cross-country comparisons. Future work will be required to update and expand on the range of services covered and to incorporate new data and methodological developments in cost estimation as they become available.

## 1 Introduction

### 1.1 Overview

This paper presents a set of unit costs for non-acute care services in Ireland for the years 2016—2019 ^1^.

A unit cost refers to the value of resources used to produce a single good or service ( Creese & Parker, 1994). For example, in healthcare, a unit cost can refer to the cost of a general practitioner (GP) visit, an outpatient appointment, a laboratory test, an episode of inpatient hospital care, a week in a nursing home etc. ( Conteh & Walker, 2004; Curtis & Burns, 2019). Unit costs are important data inputs for assessments of health service performance and value for money ( Curtis & Burns, 2019).

Internationally, while some countries have an established database of unit costs for healthcare (e.g., the UK), the need for a more coordinated approach to calculating healthcare unit costs has been recently highlighted ( Mayer *et al.*, 2020a). In Ireland, detailed cost analysis of acute care is undertaken by the Healthcare Pricing Office (HPO) but there is no central database of unit costs for community-based non-acute healthcare services ( Whyte *et al.*, 2018). This paper seeks to address this gap and presents unit costs for 16 healthcare professionals including publicly employed allied healthcare professionals, public health nurses, and a range of health care assistant staff, as well as GPs, dentists, and public and private long-term residential care (LTRC) for the years 2016–2019. It is important to acknowledge at the outset that this is a first step towards establishing a central database of unit costs for non-acute healthcare services in Ireland and future work will be required to update and expand on the range of services covered and to incorporate new data and methodological developments in cost estimation as they become available. The work presented here is the result of ongoing consultation with an advisory group and other stakeholders, drawing on their expertise in order to adopt a consensus approach in the decisions around prioritising data collection and in shaping the methodology.

In this introduction we discuss first the rationale for developing a set of unit costs for non-acute care ( section 1.2 and section 1.3), examine background literature on established methods for estimating unit costs ( section 1.4), and provide a brief overview of international and national guidelines for, and examples of, healthcare unit cost databases ( section 1.5 and section 1.6).

### 1.2 Why unit costs for non-acute care in ireland?

Resource allocation decisions in healthcare rely on valid and consistent data (e.g., on efficacy, efficiency, equity) including good cost data based on “methodologically sound unit cost information” ( Mayer *et al.*, 2020a:1142). Unit costs are used in a range of health economic studies including cost-of-illness studies, economic evaluations of alternative healthcare services and technologies, budget impact analyses, and other costing studies. Unit costs are also required for projection models that estimate future demand for, and cost of, healthcare services; for example, the Hippocrates projection model of Irish healthcare demand and expenditure ( Wren *et al.*, 2017).

In Ireland, the absence of a national unit cost database is frequently mentioned in the Irish health economic literature (e.g., Gillespie *et al.*, 2019) and the disadvantages include inconsistencies across studies with potential for misinterpretation of cross-study comparisons, challenges in making international comparisons because of inconsistent/incomplete reporting of methods and time-consuming and costly duplication of efforts by researchers to estimate unit costs.

The development of activity-based funding for acute public hospital care by the HPO has improved the availability of consistent unit cost estimates for inpatient and day cases in public hospitals. Inpatient and day case hospital costs are calculated using detailed bottom-up methods and there are plans to expand activity-based funding to acute outpatient services ^2^. Several Irish costing studies have used the casemix costs published by the HPO (e.g., Butler *et al.*, 2016; Connolly *et al.*, 2015; O'Sullivan *et al.*, 2016).

Many Irish costing studies also incorporate costs of community-based non-acute services in their analysis, from cost-of-illness studies (e.g., stroke costs, Smith *et al.*, 2012; dementia costs, Connolly *et al.*, 2014, costs of managing wound-care, Gillespie *et al.*, 2019), to economic evaluations (e.g., palliative care, Brick *et al.*, 2015, extending HPV vaccination to boys, HIQA, 2018a). However, there is no single repository of unit costs for these non-acute services and researchers are required to draw on multiple data sources and methods.

Moreover, recent analysis has shown that non-acute care has a very uneven distribution across the country with considerable geographic inequity in supply ( Smith *et al.*, 2019). Further investment in the non-acute care sector has been acknowledged as an important priority in the Sláintecare reform programme if more care is to be delivered, where appropriate, in non-acute rather than acute settings ( Government of Ireland, 2018; Houses of the Oireachtas Committee on the Future of Healthcare, 2017). Identifying unit costs for non-acute services will also facilitate research on how non-acute care can integrate with, or substitute for, acute care, which are important questions for the current reform programme.

Thus, this paper aims to provide a set of unit costs for a range of community-based non-acute services using standardised and transparent methods for use in health costing studies in the Irish context.

At the outset, there are important contextual factors that need to be taken into account in the development of these unit costs.

- First, public service salaries have undergone several changes over recent years and thus it is useful to look at the trends in unit costs. This paper presents unit costs for the years 2016–2019. The economic recession of 2008/2009 led to public service salary reductions and other cost saving measures (e.g., increased working hours and reductions in annual leave), detailed in the Financial Emergency Measures in the Public Interest (FEMPI) Acts ( Government of Ireland, 2009a; Government of Ireland, 2009b; Government of Ireland, 2013), public service agreement ( DPER, 2010), and national recovery plan ( Government of Ireland, 2010). In the subsequent years, as the economy started to recover, public service salaries have been revised upwards at different time points, sometimes mid-year, and these adjustments are taken into account in the unit cost calculations.
- Second, the analysis for this paper has taken place in the context of the coronavirus disease 2019 (COVID-19) pandemic, resulting in an inevitable interruption to normal channels of communication with, and data access from, the Health Service Executive (HSE) and other government departments. There are some data gaps and these are highlighted where relevant. As mentioned above, the set of unit costs included in this paper should be seen as a fluid set of data where improvements can, and should, be made over time if and when more resources are put into establishing and maintaining an Irish database of healthcare unit costs. Moreover, unit costs for additional services should be added over time. A key advantage of this paper is that costing studies in the Irish context will have a single port of call for a range of unit costs for non-acute care thereby ensuring consistency and transparency in methods making cross-study and cross-country comparisons easier in the future.

### 1.3 Which unit costs?

For the purposes of this paper, “non-acute” refers to care services provided outside of an acute hospital setting (e.g., in a health center, home, long-term residential care facility). Healthcare professionals working in community-based non-acute settings can include GPs, allied health professionals, nurses, dentists, and a range of health care assistants. These professionals include private practitioners (e.g., GPs) and publicly employed professionals.

The recent geographic profile of non-acute supply in Ireland provides a useful overview of the non-acute care sector, focusing on the most central non-acute healthcare services in Ireland, representing the key professions that make up primary care teams and wider community healthcare networks ( Smith *et al.*, 2019; Wren *et al.*, 2017).

The analysis by Wren *et al.* (2017) and Smith *et al.* (2019) provide a starting point for identifying which services to focus on for the development of a set of unit costs for non-acute care, namely allied health, public health nursing, GP care, and LTRC. In addition, this paper includes unit costs for a range of health care support staff (see Table 1 for the full list of unit costs) and dentists. The majority of unit costs are for publicly employed or publicly financed services, with private fees for GPs, Dentists, and LTRC also included.

### 1.4 Unit cost methods

There are two broad methodological approaches for estimating unit costs in healthcare: the top-down approach (also known as the step-down, gross costing or average costing approach) and the bottom-up approach (encompassing micro-costing, activity-based costing, and patient-level costing) ( Batura *et al.*, 2014; Olsson, 2011; Whyte *et al.*, 2018). There are advantages to each and it is generally accepted that different costing methods are appropriate depending on the specific service under investigation ( Mayer *et al.*, 2020a).

The top-down methodology assigns aggregated healthcare expenditure to individual units based on some measure of use ( Chapko *et al.*, 2009) (e.g., total hospital outpatient expenditure divided by the total number of outpatient consultations to give a cost per consultation). This approach assumes that costs are equally distributed across patients and can be suitable for relatively homogenous services that have relatively similar material and personnel use and similar utilisation patterns across patients ( Beecham, 1995; Edbrooke & Hibbert, 1999; Mogyorosy & Smith, 2005; Oostenbrink *et al.*, 2002; Waters & Hussey, 2004). The approach is less suitable for complex services where there is considerable variation in resource type and/or intensity of use across patients (e.g., hospital inpatient care) ( Whyte *et al.*, 2018). A more detailed top-down approach has also been developed whereby patients with shared characteristics are divided into sub-groups so that an average cost is generated for each sub-group ( Whyte *et al.*, 2018).

In the bottom-up methodology, and in particular, micro-costing, each component of resource used to produce a given service (e.g., staff, equipment, office space) is identified, measured, valued, summed and then divided by a specific unit of analysis (e.g., per hour, per contact) to give the unit cost ( Mayer *et al.*, 2020a). For example, a visit to a physiotherapist in a local health center is likely to require several resource components including direct (i.e., physiotherapist) salary costs, indirect salary costs (e.g., health center office staff, cleaning, etc.), indirect administrative costs (e.g., lighting, heating, office supplies), capital overhead costs (i.e., building costs), and others. These individual resource components are identified, measured, valued, summed and then divided by an agreed metric, such as annual working hours, to give a unit cost per hour, or annual number of patient contacts, to give a unit cost per contact, and so on.

Bottom-up costs can be more precise than costs based on the top-down methodology and they also allow for greater analysis of variations in costs across patients, but are more data and time intensive ( Cunnama *et al.*, 2016; Mogyorosy & Smith, 2005; Whyte *et al.*, 2018). In practice, many studies use a mix of top-down and bottom-up approaches depending on data availability and the importance of each cost item to the overall analysis ( Hendriks *et al.*, 2014) and this is in line with available international costing guidelines for health economic analysis ( Drummond *et al.*, 2015) as discussed in the next section.

### 1.5 Guidelines for unit cost databases

***1.5.1 International guidelines.*** Guidelines for conducting economic evaluation and other costing studies in healthcare include broad guidelines for measuring costs ( Drummond *et al.*, 2015; McPake *et al.*, 2020). For example, there are guidelines on which costs should be included in economic evaluations and these decisions are influenced by the perspective of the study (e.g., patient travel could be an important cost component where a societal perspective is adopted, but not perhaps where a provider perspective is adopted, Drummond *et al.*, 2015). There are also general guidelines on how costs should be measured (e.g., opportunity cost, market price, valuations for non-market items, etc.).

The preferred cost in economics is the opportunity cost, that is, the value of the benefit foregone when a resource is consumed and not available for its best alternative use ( McPake *et al.*, 2020). The preference for opportunity cost in health economic evaluations was recently confirmed in a Delphi study among European health economists, with country-specific standard costs being a recommended proxy measure ( Mayer *et al.*, 2020a). In a comparison of alternative methods for valuing GP care in four European countries (Austria, Germany, Netherlands, UK), Mayer *et al.* (2020a) noted that the UK unit cost for GP care most closely resembled the opportunity cost concept, consistent with a societal perspective. In contrast, the German and Dutch methods were closer proxies of the payer perspective than of a societal perspective.

However, there is general consensus in the broad costing guidelines that different unit costing approaches can be justified depending on the analytical perspective of the study. It is also acknowledged that costing exercises require considerable effort and are time-consuming and that there is a degree of judgement required to determine how precise cost estimates need to be within a given study ( Drummond *et al.*, 2015). The decision to embark on micro-costing or to use a more aggregated cost estimate for a specific service depends to a large extent on the quantitative and qualitative importance of that service to the intervention being analysed. In other words, the more important the cost item is to the analysis, the greater the effort should be made to estimate it accurately ( Drummond *et al.*, 2015; Mayer *et al.*, 2020a; McPake *et al.*, 2020).

There are also country-specific guidelines for estimating unit costs in healthcare. For example, the Canadian Agency for Drugs and Technologies in Health (CADTH) has outlined costing methods for commonly used healthcare services in Canada with some guidelines on when different costing methods are to be used ( CADTH, 2016). In Australia, the Department of Health periodically publishes a unit costs guideline for hospital and community-based services including residential and home care ( Australian Government DOH, 2016). In Europe, the PECUNIA ^3^ project was established in 2018 to ensure greater consistency and comparability of cost (and outcome) data across studies, sectors and countries. Pilot tests of preliminary standardised unit cost calculation templates have demonstrated general applicability and validity and have highlighted areas for improvement ( Mayer *et al.*, 2020b).

Internationally standardised unit cost calculation templates will assist the process of establishing databases of transparent country-specific unit costs for healthcare in European countries. In advance of these becoming finalised, this paper follows closely the methods and guidelines outlined by the Personal Social Services Research Unit (PSSRU) in the UK as well as Irish guidelines where available. The PSSRU has maintained a unit cost database for a wide range of acute and non-acute health and social care services since 1992. The PSSRU unit costs have been used extensively in costing studies and economic evaluations both in the UK and in applications to other countries including Ireland ( Whyte *et al.*, 2018).

The PSSRU outlines core requirements for unit costs and these are summarised here ( Curtis & Burns, 2019):

- Unit costs should be consistent across different economic analyses.
- Unit costs should be comprehensive and consider long-run marginal costs (e.g., staff qualification costs, building costs) as well as direct costs such as salaries.
- Unit costs should be clearly documented so that “it is clear what judgements have been made in constructing them, so that they can be used in an informed way” ( Curtis & Burns, 2019:1).

More details on the PSSRU guidelines and methods are outlined in Section 2. The next section introduces available Irish guidelines for unit cost estimation.

***1.5.2 Irish guidelines.*** The Public Spending Code (PSC) in Ireland is a set of rules and procedures that public bodies are required to follow when evaluating, planning, and managing public resources (both current and capital expenditures) ^4^. The PSC also contains detailed technical guidelines for assistance in cost-benefit analyses, regulatory impact analyses, financial assessments, etc. In particular, there are detailed descriptions of key central technical references and parameter values for use in financial and economic appraisal, including guidelines for the estimation of salary costs (encompassing pension costs and overheads) ( DPER, 2019).

The Health Information and Quality Authority (HIQA) produces healthcare-specific guidelines. These include guidelines for budget impact analyses of health technologies ( HIQA, 2018b) and for economic evaluations of health technologies ( HIQA, 2020). While there are detailed guidelines for the estimation of drug costs, and the guidelines for labour costs follow closely those outlined under the PSC, both reports note that the methods for identifying other cost data in the Irish context are not well defined ( HIQA 2018b; HIQA 2020). HIQA acknowledges that the absence of a central medical costs database in Ireland means that generating Irish healthcare cost data is “challenging and time consuming” ( HIQA, 2020:35). In this context, HIQA recommends the need for flexibility regarding cost valuation and that assumptions and cost estimates should be clearly reported and subjected to sensitivity analysis.

This paper draws on these Irish guidelines where possible to inform the methodology for estimating unit costs for non-acute healthcare, outlined in detail in Section 2. In particular, the paper draws on available Irish recommendations for estimating labour costs. However, where there is strong justification for modifying the parameters set out in the Irish guidelines (e.g., on pensions and overheads), these are discussed in detail and clearly reported. The overall aim is to use available data and methodological guidelines to develop unit costs that are consistent with the definition of opportunity costs.

### 1.6 International and national unit cost data

***1.6.1 International unit cost data.*** To assist health service performance assessment (e.g., economic evaluations, cost-of-illness studies, etc.) and ultimately to support resource allocation decisions in healthcare, some European countries (e.g., UK, the Netherlands) have developed national unit cost databases ( Mayer *et al.*, 2020a) although these vary from built-for-purpose, regularly updated databases (e.g., UK) to systematic reviews of existing cost studies (e.g., Austria).

As noted above, in the UK, the PSSRU publishes unit costs in detailed tables with transparent methods and data sources that are updated on an annual basis ( Curtis & Burns, 2019). Different methods are applied depending on the service in question ( Whyte *et al.*, 2018). Of direct relevance to this study, a micro-costing approach is used to generate unit costs for community-based healthcare professionals and this approach is described in more detail in Section 2.

In the Netherlands, costing manuals are published periodically by the Dutch National Health Care Institute (2000, 2004, 2010, and 2017) ( Kanters *et al.* 2017; Oostenbrink *et al.* 2000; Tan *et al.*, 2012). Bottom-up and top-down methodologies are applied to different healthcare services (e.g., bottom-up approach for hospital care costs, top-down approach for primary care physicians, elderly care, home care).

In Austria, the Medical University of Vienna established a unit cost library in 2016. The publicly accessible online database is a compilation of unit costs retrieved from Austrian costing studies and economic evaluations for the years 2004—2019 ( Mayer *et al.*, 2020a).

In countries where there are no established unit cost databases, there is evidence of considerable research time spent on sourcing and/or estimating unit cost data. Several costing studies and economic evaluations contain detailed methods and data on healthcare costs using both top-down and bottom-up approaches. Costs can be country-specific or area-specific, or specific to a particular disease or healthcare intervention. Recent examples of top-down analyses range from estimates of unit costs of public hospital costs in Myanmar ( Than *et al.*, 2017) to estimates of hospital and other costs in a cost-effectiveness analysis of treatments for severe gastroesophageal reflux disease (GERD) in Korea ( Park *et al.*, 2020). Recent examples of bottom-up analyses include detailed estimates of costs of community-based cardiovascular disease prevention care in the USA ( Wang *et al.*, 2019), and costs of facilitating a very early infant diagnosis (VEID) test of HIV in Lesotho ( Tchuenche *et al.*, 2018); see also a recent review of the unit cost literature by Whyte *et al.* (2018).

However, there are also many economic analyses that source unit cost information from administrative sources with little attention paid to the costing approach ( Mayer *et al.*, 2020a). Mayer *et al.* (2020a) demonstrate the importance of thinking about, and using the most appropriate, methodological approach when estimating and using unit costs. The authors applied six different unit costing methodological approaches to Austrian data on GP care and found that the cost per GP consultation varied by more than 170% across the different methods. The authors emphasise the importance of a standardised cost database for countries that do not have a set of national unit costs in order to increase the quality of cost data used in health economic analyses and also to “potentially improve the acceptability of such evidence in policy making” ( Mayer *et al.*, 2020a:1146).

***1.6.2 Irish data on unit costs.*** In the absence of a national database of unit costs for healthcare, Irish costing studies have used various data sources and methods for cost estimation and many of the costs included are specific to the healthcare service or technology under examination ( Whyte *et al.*, 2018).

However, despite the absence of a central database, researchers have applied, where possible, consistent methods and estimates for community-based non-acute care services. For example for publicly employed allied healthcare visits, many Irish costing studies reference HSE salary scale data ( Connolly *et al.*, 2014; Connolly *et al.*, 2015; Gillespie *et al.*, 2019). This indicates that the Irish guidelines on labour costs have been applied in these studies although detailed methods (e.g., pension cost, overhead estimates) are not presented. Similarly, a common estimate of the fee paid for a private GP visit has been applied in a number of studies with some limited explanation of the methods (e.g., Connolly *et al.*, 2014; Gillespie *et al.*, 2013; Smith *et al.*, 2012).

Other studies have relied on international cost estimates, for example using UK costs where no Irish data are available (e.g., Gillespie *et al.*, 2011; Gillespie *et al.*, 2019; Manca *et al.*, 2003) and authors have emphasised the need for standardised national unit costs (e.g., Gillespie *et al.*, 2019).

In a recent economic evaluation of palliative care in Ireland, the PSSRU methodology was applied to Irish data for a range of non-acute allied health services ( Brick *et al.*, 2015). Where Irish-specific data were unavailable (e.g., public sector pension rates, overhead rates), UK data were applied. In this paper, the PSSRU methodology is also applied but we expand on the approach by Brick *et al.* (2015) by applying Irish-specific data throughout (with the exception of capital costs), presenting unit costs for a wider range of services and for multiple years.

The rest of the paper is structured as follows: Section 2 outlines the methods and data used to calculate unit costs for non-acute services in Ireland. Section 3 presents unit cost tables for selected non-acute services in Ireland for the period 2016–2019. Section 4 presents some concluding comments including recommended next steps for further development of a database of unit costs for non-acute care in Ireland.

## 2 Methods and data

### 2.1 Introduction

The majority of the unit costs included in this paper are calculated using the micro-costing methodology outlined by the PSSRU. The methods are presented in detail in this section along with the data sources used to generate Irish-specific unit costs. These methods are applied to Irish data for the publicly employed healthcare professionals in community-based healthcare services in Ireland listed in Table 1.

**Table 1.** List of healthcare professionals and unit cost estimation method.

Healthcare professional	Staff Category	Staff Group	Unit cost estimation method
Dietitian	Health & Social Care	Therapies	Micro-costing (based on PSSRU methods)
Occupational Therapist	Health & Social Care	Therapies	Micro-costing (based on PSSRU methods)
Physiotherapist	Health & Social Care	Therapies	Micro-costing (based on PSSRU methods)
Podiatrist & Chiropodist	Health & Social Care	Therapies	Micro-costing (based on PSSRU methods)
Psychologist	Health & Social Care	Therapies	Micro-costing (based on PSSRU methods)
Social Care Worker	Health & Social Care	Therapies	Micro-costing (based on PSSRU methods)
Public Health Nurse	Nursing & Midwifery	Public Health Nurse	Micro-costing (based on PSSRU methods)
Attendant (Multi-Task)	Patient & Client Care	Health Care Assistants	Micro-costing (based on PSSRU methods)
Care Assistant (Disability Services)	Patient & Client Care	Health Care Assistants	Micro-costing (based on PSSRU methods)
Health Care Assistant	Patient & Client Care	Health Care Assistants	Micro-costing (based on PSSRU methods)
Health Care Support Assistant (formerly Home Help)	Patient & Client Care	Home Help	Micro-costing (based on PSSRU methods)
GP Public	n/a	n/a	Top-down method
GP Private	n/a	n/a	Survey of private fees
Dentist Public	n/a	n/a	Survey of private fees
Dentist Private			
Long-Term Residential Care	n/a	n/a	Bottom-up (public) & Negotiated fees (private)

Note: n/a = not applicable

Alternative methods are applied to generate public and private unit costs for GP care, dental care, and LTRC and these are also outlined in this section. As outlined in Section 1, these represent the most central non-acute healthcare services in Ireland ( Smith *et al.*, 2019; Wren *et al.*, 2017). All analyses are undertaken in Microsoft Excel (Version 2008).

### 2.2 Micro-costing methods

***2.2.1 Overview.*** The cost estimation approach developed by the PSSRU aims to be transparent and flexible. This paper focuses on the bottom-up approach that is used by the PSSRU to estimate unit costs for community professional staff, community-based nurses and other healthcare professionals ^5^. The approach is outlined in Table 2, giving examples of unit costs for different healthcare professionals in the UK, and shows that the unit costs are comprised of separate cost components. These components include wages, salary oncosts (encompassing employers’ national insurance and superannuation), qualification costs (where available), overheads (staff and non-staff), capital overheads, and travel (where available). Total cost (i.e. the sum of the components) is divided by the estimated number of hours worked per year to give a unit cost per hour.

**Table 2.** PSSRU methods for unit costs for selected healthcare professionals.

Unit cost components		Description	2019 costs (Band 5) £GBP	
			Community, Scientific & Professional Staff (e.g., Occupational Therapist, Speech & Language Therapist, Podiatrist)	Community-based nurses
A	Wages	Mean basic FTE salary per year	£24,212	£26,894
B	Salary oncosts	Employers’ national insurance Superannuation (14.38% of A)	£5,660	£6,416
C	Qualification costs	Estimated qualification costs	not included	£8,687
D	Overheads	Management, admin and estates staff	£7,319	£8,161
		Non-staff	£11,411	£12,363
E	Capital overheads	Based on new-build & land requirements of NHS hospital facilities	£5,237	£3,814
F	Travel	Not included (no data on mileage)		
G	Working time		42.6 weeks (1,599 hours) per year 37.5 hours per week	41.9 weeks (1,573 hours) per year 37.5 hours per week
Cost per working hour		(A+B+C+D+E+F)/Working time	£34 (excluding qualifications) per hour	£37 (excluding qualifications) per hour

Notes: PSSRU = Personal Social Services Research Unit; FTE = full time equivalent Source: Table adapted from Curtis & Burns, 2019

Where a user has more up-to-date information on a specific cost component they can substitute their own data for any component ( Curtis & Burns, 2019), and this lends flexibility to the unit costs. Sources of information are provided for each cost component thereby ensuring transparency ( Curtis & Burns, 2019).

Each of the cost components in Table 2. is described in more detail in the following sections. For each component, the availability of Irish data is also outlined in order to generate Irish-specific unit costs for the healthcare professionals listed in Table 1. Irish unit costs are calculated for each healthcare professional for each year in the period 2016–2019 inclusive.

***2.2.2 Cost component A: wages/salary***

**2.2.2.1 Selection of grade code**. The first component in the micro-costed unit cost is the annual salary of the healthcare professional.

In the Irish context, the first decision to make for each healthcare professional is which grade code to focus on. Publicly employed HSE staff are categorised as follows:

- Six Staff Categories: Medical & Dental; Nursing & Midwifery; Health & Social Care Professionals; Management & Administrative; General Support; Patient & Client Care
- These categories are further subdivided into 26 Staff Groups, 84 Grade Groups and 600 Grades.

Each Grade is given a grade code which is linked with a Grade Group, Staff Group and Staff Category. In line with the approach adopted by Brick *et al.* (2015) for generating unit costs for palliative care, we have selected the grade code with the largest number of whole-time equivalents (WTEs) employed, in other words, the modal group. For example, Table 3 outlines the percentage breakdown of WTE community-based OTs by grade code based on data from the Health Service Personnel Census, HSE. The majority of WTE OTs employed in community services are working at the grade of Senior OT. Thus, the unit cost for OT in this paper is based on the salary that applies to the grade of Senior OT.

**Table 3.** Percentage of publicly employed, community-based, WTE Occupational Therapists, by grade code and year.

Grade code	2016	2017	2018	2019	2020
Manager	7%	7%	7%	7%	na
Specialist	0%	0%	0%	0%	na
**Senior**	**54%**	**55%**	**55%**	**55%**	na
Basic	39%	39%	38%	37%	na
Total	100%	100%	100%	100%	na

Notes: For ease of presentation these categories group together some specific grade codes. The number of grade codes in each category varies by healthcare professional. For example, for OT, there is one grade code in the basic category, where the highest point on the salary scale for OT (€52,970 in 2019) is close to the starting salary point for OT, Senior in the next category (€53,074 in 2019) ( HSE, 2020a) .WTE = Whole Time Equivalent; na = not available Source: Health Service Personnel Census, HSE.

Economic evaluations and other costing studies that apply unit costs to utilisation rates typically rely on high-level data on utilisation (e.g., number of physiotherapy visits, number of OT visits, number of GP visits), rather than on detailed utilisation disaggregated by grade code (e.g., number of visits with an OT; number of visits with an OT, Senior; number of visits with an OT, Manager). In the absence of utilisation data disaggregated by grade code, assumptions need to be made as to which grade codes, and their associated costs, to focus on. Thus, this paper aims to assist this process by identifying, and calculating the unit costs for the modal grade codes based on a reasonable working assumption that these are the most likely grade codes that patients encounter in community-based services. However, it is important to note that the same methods for calculating unit costs that are outlined here can be applied to any grade code. The HSE consolidated salary scales are publicly available ( HSE, 2020a) if unit costs for alternative grade codes are needed.

For the majority of healthcare professionals included in this section of the paper, one grade code accounts for 50–100% of the WTEs working in community services.

**2.2.2.2 Basic salary or total earnings**. The second decision to make regarding the salary component in the micro-costed unit cost is whether to include basic salary or total earnings.

Basic salary refers to the contracted salary before any additional allowances and other benefits (in cash/in kind) are added. Total earnings refer to the total payments made after allowances and other benefits are added to basic salary. For HSE employees, these additional payments can include payments for overtime, on-call work, allowances, weekend and night-duty work, and any other arrears ( Brick *et al.*, 2015).

Basic salary has the advantage of being both publicly available ( HSE, 2020a) and consistent with the PSSRU approach and is adopted for this analysis ^6^. Alternatively, examination of earnings would give a more accurate picture of the total HSE staffing costs of running public community-based services. However, it is more difficult to calculate the average earnings for a given grade and would require additional analysis in conjunction with the HSE but this should be considered for future analysis of unit costs.

Basic salary scales for each grade code are available from the HSE for each of the years included in this paper ( HSE, 2020a). Where salaries are revised mid-year, a weighted average salary for the year is calculated, as described below.

Following the recession of 2008/2009, as the economy began to recover, salaries to public servants were revised upwards on a number of occasions over the period 2016—2020. Some of these revisions occurred mid-year. For example, on 1 ^st^ January, 2019, salaries for public servants earning less than €30,000 were increased (by 1%) in line with requirements of the Public Service Pay and Pensions Act 2017 ( Government of Ireland, 2017). On 1 ^st^ April, 2019, salaries for higher earning public servants were increased to restore pay to pre-recession levels in line with the FEMPI Act 2015 ( Government of Ireland, 2015), and on 1 ^st^ September, 2019, salaries for all public servants were increased by 1.75% in accordance with the Public Service Pay and Pensions Act 2017 ( Government of Ireland, 2017). Where the salary for a given grade code is revised mid-year, a weighted average salary for that year is calculated based upon the month where the revision was implemented. This is illustrated in Equation 1 using the example of OT, Senior in 2019 where the salary (at point 5, the mid-point of the scale) was revised from €56,695 to €57,687 on the 1 ^st^ September of that year.

$$\text{Equation}\;1:\text{OT},\;\text{Senior}\;(\text{salary}\;\text{point}\;5):\;€56,695\times \left(\frac{8}{12}\right)+€57,687\times \left(\frac{4}{12}\right)=€57,025$$

For each grade code, there are different salary points and the number of points varies by grade code. Employees move up these points on an annual basis subject to performance assessment by the HSE ^7^. The salary at the mid-point of the scale is used for the calculation of the unit cost in line with HIQA guidelines for budget impact and economic evaluations and Department of Public Expenditure and Reform (DPER) guidelines for economic appraisals ( DPER, 2019; HIQA, 2018b; HIQA, 2020). Where there is an even number of salary points (e.g., 8), the mean of the two middle points is calculated (e.g., mean of points 4—5). For the purposes of this analysis, long service increments (LSIs), where applicable, are counted as normal points on the salary scale, in line with DPER guidelines on salary calculations ( DPER, 2019). LSIs are additional salary points that apply when the employee has reached the top of the salary scale and has remained there for a specified number of years. For example, the first LSI applies after 3 years at the top of the normal salary scale. The second LSI applies after 3 years on LSI1 and so on ( HSE, 2020a).

***2.2.3 Cost component B: salary oncosts***

**2.2.3.1 Pay Related Social Insurance calculation**. Pay Related Social Insurance (PRSI) is payable by employees and employers. Employer PRSI contribution is included in the unit costs as part of ‘salary oncosts’. PRSI contribution rates are categorised into different classes (A, B, C, D, E, H, J, K, M, S, and P) although the majority of employees are liable to pay Class A PRSI ( DEASP, 2020). It is assumed that the healthcare professionals included in the unit cost database are in Class A in line with HIQA and DPER guidelines ( DPER, 2019; HIQA, 2018b; HIQA, 2020). Class A is divided into sub-classes based on weekly pay bands and the contribution rates vary across these bands (see Table 4). For each healthcare professional, the annual mean basic salary (see above) is divided by 52 (i.e., to give weekly salary) to determine the appropriate weekly PRSI rate. The appropriate PRSI rate is multiplied by the annual mean basic salary to give the annual employer PRSI contribution.

**Table 4.** PRSI rates for Class A, 2016—2020.

Class A	2016		2017		2018		2019		2020	
Subclass	Weekly pay band	Employer rate %	Weekly pay band	Employer rate %	Weekly pay band	Employer rate %	Weekly pay band	Employer rate %	Weekly pay band ^a^	Employer rate %
A0	€38 - €352	8.5	€38 - €352 inclusive	8.5	€38 - €352 inclusive	8.6	€38 - €352 inclusive	8.7	€38 - €352 inclusive	8.8
AX	€352.01 - €376	8.5	€352.01 - €376 inclusive	8.5	€352.01 - €376 inclusive	8.6	€352.01 - €386 inclusive	8.7	€352.01 - €395 inclusive	8.8
AL	€376.01 - €424	10.75	€376.01 - €424 inclusive	10.75	€376.01 - €424 inclusive	10.85	€386.01 - €424 inclusive	10.95	€395.01 - €424 inclusive	11.05
A1	More than €424	10.75	More than €424	10.75	More than €424	10.85	More than €424	10.95	More than €424	11.05

Notes:     PRSI = Pay Related Social Insurance ^a^From February 2020 onwards Sources: Department of Social Protection, 2016; Department of Social Protection, 2017; DEASP, 2018; DEASP, 2019; DEASP, 2020

**2.2.3.2 Superannuation**

*Irish guidelines on public sector pension costs*

Employer contribution to superannuation is included in the unit costs as part of the salary oncosts.

Healthcare professionals employed by the HSE are eligible for a public sector pension. The full cost of a public sector pension is difficult to identify *ex ante* because the Government operates a ‘pay as you go’ scheme whereby public sector retirement benefits are paid as and when the costs arise. In contrast, private sector pension costs are much more visible because the pension benefits must be funded in advance via employee and employer contributions to a pension fund ( DPER, 2017a).

HIQA guidelines for budget impact and economic evaluations of health technologies suggest that a pension rate of 4% (of basic salary) captures the employer contribution to superannuation, citing 2012 guidelines from DPER as the source ( HIQA, 2018b; HIQA, 2020).

More recent DPER guidelines for economic appraisals ( DPER, 2019) recommend higher pension rates based on an actuarial review of the cost of public sector pensions ( DPER, 2017a). The review estimated a notional contribution, expressed as a percentage of pensionable salary, that would be required to be paid throughout the working life of an average employee in order to generate the pension (lump sum plus gratuity) owing to that employee ( DPER, 2017a). Estimated employee pension contributions were deducted from this notional contribution to give the net value of the Government’s contribution to the employee’s pension.

Benefit regimes for public sector pensions have been adjusted over time and there are different cohorts of pension scheme members depending on their date of joining the public service. The most notable difference is between members who joined the public service prior to 2013, and those who joined on or after 1 ^st^ January 2013 when the Single Public Service Pension Scheme (the “Single Scheme”) was introduced. The benefit regime in the Single Scheme has a very different structure compared with pre-2013 public sector pensions (e.g., retirement benefits are based on career average pensionable salary rather than final salary; later minimum pension age; Consumer Price Index linking rather than pay parity increases).

Thus, the actuarial review reported different notional employer pension contribution rates for pre- and post-2013 entrants to selected posts such as nurses, as well as an overall average contribution rate for public sector posts that have broadly similar benefit structures and salary progression (including Civil Servants, National School Teachers, Nurses, and Engineers), see Table 5.

**Table 5.** Notional employer pension contributions as percent of salary, 2017 (excluding PRD).

	Pre-2013 entrants	Post-2013 entrants
Nurse	28%	8%
Civil Servant	27%	8%
National School Teacher	29%	9%
Engineer	33%	10%
DPER Average	29%	9%

Note: PRD = Pension Related Deduction Sources: DPER, 2017a; DPER, 2019

The figures in Table 5 do not take into account the Pension Related Deduction (PRD). The PRD was a deduction from the salary of pensionable public service employees between 2009 and 2018 as part of the wider package of emergency financial measures aimed at stabilising public finances following the economic recession of 2008/2009 ( DPER, 2017a). Inclusion of PRD rates would reduce the notional employer contribution but these were not applied to the calculation of net notional employer contribution rates in the 2017 review because the PRD was not considered a pension contribution under legislation ( DPER, 2017a).

However, the PRD has since been replaced by the Additional Superannuation Contribution (ASC) which is a permanent pension contribution linked with legislation ( DPER, 2017b; Government of Ireland, 2017). Thus, although the PRD was a temporary measure, it was replaced rather than discontinued and it is important to take the PRD and its successor, the ASC, into account in order to calculate a more realistic estimate of the notional employer public pension contribution rate.

The DPER actuarial review estimated that the average rate of PRD for the posts included in Table 5 was 5%. Taking PRD into account for the years 2016—2018 would reduce the average notional employer contribution rate to 24% (pre-2013 cohort) and 4% (post-2013 cohort) ^8^. The ASC rates for 2019 and 2020 are lower than the PRD rates, particularly for the post-2013 cohort. For 2019, the average ASC rates are estimated to be 4% and 2% for pre-2013 and post-2013 cohorts respectively, bringing the average notional employer contribution rates to 25% (pre-2013) and 7% (post-2013) respectively ^9^. The notional employer contribution rates for 2016—2020 with and without the PRD/ASC are outlined in Table 6.

**Table 6.** Notional public sector employer contribution rates 2016—2020 with PRD/ASC.

	Employer contribution rate (%)									
	Pre-2013 cohort					Post-2013 cohort				
	2016	2017	2018	2019	2020	2016	2017	2018	2019	2020
**Without PRD/ASC**										
DPER average rate	29	29	29	29	29	9	9	9	9	9
Nurse	28	28	28	28	28	8	8	8	8	8
**With PRD/ASC**										
DPER average rate	24	24	24	25	26	4	4	4	7	8
Nurse	23	23	23	24	25	3	3	3	6	7

Notes: PRD = Pension Related Deduction; ASC = Additional Superannuation Contribution; DPER = Department of Public Expenditure and Reform Sources: DPER, 2017a; DPER, 2017b; Government of Ireland, 2017; DPER, 2019

In this analysis, the pension rates with PRD/ASC that are outlined in Table 6 are adopted, combined with assumptions about the proportion of health service employees in each of the pre- and post-2013 cohorts.

*Pension cohorts within HSE*

Application of the separate pre- and post-2013 DPER public sector pension employer contribution rates in the unit costs for publicly employed community-based staff requires data on the proportion of HSE employees in each pension cohort.

Data on the number of active HSE (including Section 38) Single Scheme members at December of each year are deducted from the total number of HSE (including Section 38) employees to estimate the proportion of employees in the pre- and post-2013 pension cohorts for the years 2016–2019. Data on Single Scheme membership were provided by the DOH ^10^ and DPER ^11^. HSE headcount data for the years 2016–2019 were also provided by the DOH ^12^.

The pre- and post-2013 employer pension contribution rates (with PRD/ASC) from Table 6 are weighted by the estimated proportion of HSE staff in each cohort, to give the overall average pension contribution rate, presented in Table 7. The DPER average rate is used for the Health & Social Care and Medical & Dental staff categories, while the DPER nurse rate is applied to Nursing & Midwifery and Patient & Client Care to reflect different earnings trajectories.

**Table 7.** Estimated average public sector employer contribution rate 2016—2020 (with PRD/ASC), by staff category.

Staff category	Average employer pension contribution rate %			
	2016	2017	2018	2019
DPER Average	19.4	18.2	17.1	18.1
Medical & Dental	19.4	18.2	17.1	18.1
Nursing & Midwifery	18.4	17.2	16.1	17.1
Health & Social Care	19.4	18.2	17.1	18.1
Patient & Client Care	18.4	17.2	16.1	17.1

Note: PRD = Pension Related Deduction; ASC = Additional Superannuation Contribution; DPER = Department of Public Expenditure and Reform Sources: DPER, 2017a; DPER, 2017b; Government of Ireland, 2017; DPER, 2019; Personal communication with Irish Government Economic and Evaluation Service (IGEES), DOH, 04/01/2021; Personal communication with IGEES, DOH, 22/01/2021.

***2.2.4 Cost component C: qualifications.*** The inclusion of qualification costs is particularly important for analyses of changes in service delivery that involve changing the staff mix ( Curtis & Burns, 2019). For example, in the UK, including estimated qualification costs can increase unit costs for allied health professionals by 8–10% (for Bands 5–6) ( Curtis & Burns, 2019). Calculation of qualification costs would have required extensive analysis that was beyond the resources available to this project but should be considered in future unit cost estimation work in the Irish context.

***2.2.5 Cost component D: overheads***

**2.2.5.1 Irish guidelines on overheads**. Overhead costs refer to the costs of running the background infrastructure within which the healthcare professionals work. These can include non-pay costs such as utilities (e.g., light, heat, telephone, internet), accommodation costs, office facilities, and general supplies as well as administrative and management staff costs.

HIQA guidelines for budget impact and economic evaluations of health technologies suggest that an overhead rate of 25% (of basic salary) can be applied as a general rule of thumb in the absence of more detailed information on overheads ( HIQA, 2018b; HIQA, 2020). This estimate covers “rent, light and heat, office facilities, telephone, general supplies, and so on” ( HIQA, 2020: 66). The 25% estimate is taken from the well-cited textbook on methods for conducting economic evaluations by Drummond *et al.* (2015).

DPER also issues guidelines for economic appraisals and indicates that an overhead rate of 25% can be applied to cover costs of “accommodation, utilities, support and back-office staff, training, travel, etc.” ( DPER, 2019: 8) although no source is documented for this estimate ^13^.

In both cases, HIQA and DPER acknowledge the importance of using more detailed estimates of overhead costs if available ( DPER, 2019; HIQA, 2018b; HIQA, 2020). However, there are no published estimates of the overhead costs associated with professionals working in non-acute healthcare settings (e.g., health centers) in Ireland.

**2.2.5.2 PSSRU overhead estimates**. The rate of 25% for overheads is low compared with estimates used by the PSSRU. The PSSRU overhead rates for community-based professionals are based on 2013/2014 financial accounts for 10 community trusts ( Curtis & Burns, 2019) and include management and other non-care staff costs (e.g., administration) at 24.5% of salary costs (basic plus oncosts) plus non-staff costs at 38.2% of salary costs (basic plus oncosts). The non-staff costs include costs for “office, travel/transport, publishing, training courses and conferences, supplies and services (clinical and general), and utilities such as water, gas and electricity” ( Curtis & Burns, 2019: 112). Together these indicate an overhead rate of more than 60% of salary costs (basic plus oncosts). Previous application of PSSRU methods to Irish unit costs adopted these overhead rates ( Brick *et al.*, 2015).

**2.2.5.3 Selection of overhead rate and sensitivity**. The HIQA/DPER description of overheads does not include management staff and this may account for some of the difference between the HIQA/DPER and PSSRU overhead estimates. However, the current HIQA overhead rate is also lower than what was previously recommended for staff cost calculations in Ireland. The HIQA guidelines indicate that the non-pay overhead costs should be estimated ‘in accordance with the methods outlined in the Regulatory Impact Analysis (RIA) guidelines issued by the Department of the Taoiseach” ( HIQA, 2020: 66). The guidelines from the Department of the Taoiseach were issued in 2009 and recommended an overhead rate of 40% of basic salary, although no source was given for the estimate ( DOT, 2009).

There is a clear gap in evidence around the overhead costs for community-based services in the Irish setting. The expansion of activity-based funding to acute outpatient services by the HPO could produce useful data on cost breakdowns in outpatient departments in due course although this will still be second-best to detailed cost breakdowns for services in the community setting.

Given the uncertainty around the overhead estimates, three overhead scenarios are drawn from the available Irish guidelines and PSSRU data to undertake some sensitivity analysis:

-   Baseline overheads: 40% of basic salary ( DOT, 2009)

-   Low overheads: 25% of basic salary ( DPER, 2019; HIQA, 2018b; HIQA, 2020)

-   High overheads: 62.6% of basic salary + salary oncosts ( Curtis & Burns, 2019)

***2.2.6 Cost component E: capital overheads.*** The HIQA and DPER guidelines on overheads do not refer to capital costs so these are estimated from the PSSRU unit cost database. Capital costs for community-based healthcare professionals included in the PSSRU unit cost reports ( Curtis & Burns, 2018; Curtis & Burns, 2019) are calculated as a percentage of total salary costs (i.e., basic plus oncosts) and the average rates are applied to Irish salary data to give Irish capital cost estimates.

***2.2.7 Cost component F: travel.*** Similar to qualification costs, estimation of travel costs for publicly employed community-based health professionals in Ireland would have required extensive analysis that was beyond the resources available to this project and there are no up-to-date estimates from the PSSRU for these professionals ( Curtis & Burns, 2019), but these costs should be considered in future analysis.

***2.2.8 Number of hours worked per annum.*** For a given publicly employed healthcare professional, each of the cost components outlined above are summed and divided by the estimated total number of hours worked per annum to give the unit cost per hour of service. The total number of hours worked per annum is calculated as follows: estimated total number of days worked per annum (number of working days in a given year minus annual leave entitlement and estimated sickness absence days) multiplied by the number of hours worked per day. Table 8 outlines the data sources for each of these elements using the example of Occupational Therapist.

**Table 8.** Calculation of hours worked per annum, Occupational Therapist, 2019.

Days/Hours	2019	Notes & Sources
Number of working days per year	252 days	The number of working days in a given year is calculated by deducting total weekend days in that year from 365 days (or 366 in a leap year) ( DPER, 2019). For example, 2016 was a leap year and there were 105 weekend days giving a total of 252 working days in that year.
Deductions: Annual leave entitlement, estimated sickness absence days	36 days	The total number of days worked per annum is calculated by deducting the professional’s annual leave entitlement and estimated sickness absence days from the number of working days in a given year. Annual leave entitlement for selected grade codes (e.g., allied health professionals) are detailed by the HSE ( HSE, 2009a; personal communication with HSE, 04/02/2021). Leave entitlements for nurses and other grades (e.g., social care workers) are covered by national agreements ( HSE, 2014) and a number of data sources have been used to identify leave entitlements for these grades including the Irish Nurses and Midwives Organisation ( INMO, 2020). Deduction of sickness absence days is consistent with the PSSRU methodology whereas the HIQA and DPER guidelines on economic evaluations do not make any provision for sickness absence days. Brick *et al.* (2015) estimated the number of sickness absence days based on the national target for sickness absence of 3.5%. The PSSRU estimates the number of sickness absence days using data from the National Health Service (NHS) on sickness rates for different staff categories ( Curtis & Burns, 2019). In this database, absenteeism rates for HSE staff working in community services, disaggregated by staff category, are applied to total working days after annual leave days have been deducted ( HSE, 2017; HSE, 2018; HSE, 2019a; HSE, 2019b) ^14^.
Total days worked per annum	216 days	
Total hours worked per annum	1,601 hours	The number of hours worked per week are based on specified contracted hours. Working hours for public servants were revised most recently in the 2013 Public Service Stability Agreement (the ‘Haddington Road Agreement’) with the standard working week for public servants at 35 hours per week increasing to 37 hours (e.g., allied health professionals, HSE, 2009b), and for those on 37 hours per week increasing to 39 hours (e.g., nurses and midwives) ( Labour Relations Committee, 2013) ^15^. The number of hours worked per week are divided by 5 to give the total number of hours worked per day for each grade code. The number of days worked per annum are multiplied by the number of hours worked per day to give the total number of hours worked per annum. The estimated total number of hours worked per annum are assumed to be standard across all employees within the selected grade code.

***2.2.9 Ratio of direct to indirect time.*** There are some data on time use by healthcare professionals in the UK and Ireland but they are typically out of date and/or based on small sample sizes. For these reasons they are not included in this paper in line with the PSSRU approach of focusing on data sources that are less than 10 years old ( Curtis & Burns, 2019). However, the reader is directed to section V in the unit cost report by Curtis & Burns (2019) and to Brick *et al.* (2015) for further discussion of time use patterns by community-based allied health professionals and nurses (including palliative care nurses).

### 2.3 Non-micro costing methods

***2.3.1 GP unit costs.***

**2.3.1.1 Structure of General Practice in Ireland**. GPs in Ireland are self-employed private practitioners and are free to set their own prices for services provided to private patients ( Connolly *et al.*, 2018).

Many GPs hold a General Medical Services (GMS) contract with the HSE to provide GP care that is free at the point of use to Medical Card (MC) holders ( HSE, 1989) and to GP Visit Card (GPVC) holders ( HSE, 2005). MCs and GPVCs are granted mainly on the basis of an income-based means test but some are also granted on a discretionary basis where paying for healthcare would cause undue financial hardship. Since 2015, GPVCs are available for adults aged 70+ years. GPs can also hold the ‘Under 6s’ contract to provide care that is free at the point of use to children under the age of 6 years ( HSE, 2015). GPs receive an annual capitation payment (adjusted for age and sex) for each MC and GPVC holder on their list, as well as fees for out-of-hours and special items of service provided to MC and GPVC holders (e.g., excisions, sutures, etc.). A standard capitation fee of €125 and a separate schedule of fees for special items of service is payable in respect of Under 6 GPVC holders.

A range of allowances for practice support (e.g., Practice Nurse, practice secretary), rural practice supports, annual leave, study leave, sick leave, maternity/paternity/adoptive leave ( PCRS, 2019) are also available to GPs holding a GMS contract ^16^.

The capitation rates, fees, and allowances have been revised periodically but most recently the rates have been adjusted as part of a new agreement between the Department of Health (DOH), the HSE and the Irish Medical Organisation (IMO) regarding service development ( DOH *et al.*, 2019), described in more detail below.

GPs (both GMS and non-GMS GPs) are also paid fees for services delivered under specific schemes including the Primary Childhood Immunisation Scheme, National Cancer Screening Service (e.g., cervical screening), the Opioid Substitution Treatment Scheme, Heartwatch, the Health (Amendment) Act 1996, and the Maternity & Infant Care Scheme.

The majority of GPs operate in multi-person practices and many now employ a practice manager, a Practice Nurse, and other support staff ( O'Kelly *et al.*, 2016).

**2.3.1.2 Methods for GP unit costs**

*Background literature on measuring GP costs in Ireland*

Given that both public (i.e., MC and GPVC holders) and private patients are treated in private GPs practices, the most obvious approach would be to estimate a single unit cost for GP care to reflect the average cost of all (i.e., public and private) GP care across GP practices.

However, the bottom-up micro-costing PSSRU method has not been applied to GP unit costs in Ireland given the difficulty in obtaining accurate and reliable estimates of GP earnings as well as detailed estimates of the average overhead and capital costs of running a GP practice.

Rather, there are detailed data on HSE payments to GPs for treatment provided to public patients (including practice support payments), and survey estimates of average payments made to GPs for a private GP visit. Thus, there are estimates of:

-   the average cost (to the HSE) of a public GP visit;

-   and the average price of a private GP visit.

The general approach to estimating the cost of publicly funded GP care in Ireland has been to include capitation (mean, or age and sex specific) plus mean additional payments to GPs in respect of MC and/or GPVC holders. There are variations in which additional payments are included depending on the focus of the study.

For example, the most recent estimates of the costs of public GP care have focused on the marginal costs of extending eligibility for publicly funded GP care. Wren *et al.* (2015) examined the costs of introducing universal health insurance in Ireland, including estimates of the additional costs of providing universal access to GP care free at the point of use. Connolly *et al.* (2018) expanded on this analysis to focus on how to set a reimbursement price for universal GP care in Ireland and estimated the impact of universal GP care on healthcare expenditure in Ireland. In both pieces of work, the authors examined alternative remuneration rates that would be paid to GPs for existing non-cardholders in the event of universal access to publicly funded GP care. The remuneration rates applied to non-cardholders included:

- Age and gender-specific GMS capitation rate plus the average payment made to GPs in respect of existing cardholders for out-of-hours and special service fees. This scenario assumed that the remaining payments made by the HSE under the GMS contracts (e.g., secretarial and nursing payments, annual leave, rostering, rural practice, study leave, etc.) are related to practice overheads and would not apply for marginal additional payments ( Wren *et al.*, 2015).
- Age and gender-specific GMS capitation rate plus the average payment made to GPs in respect of existing cardholders for out-of-hours and special service fees, secretarial/nursing, annual leave, rostering/out-of-hours allowances, and superannuation ( Connolly *et al.*, 2018). Other fees (such as dispensing and asylum seekers) were excluded on the basis that they are specific to certain practices and do not extend to the total population ( Connolly *et al.*, 2018). Average payments under the Maternity & Infant Care Scheme were also included.
- Recent analysis by Prior *et al.* (2019) proposed a framework for examining the cost implications of changes in eligibility for a GPVC, either through granting universal eligibility to specific age cohorts, or by adjusting GPVC means assessment thresholds. Similar to the work by Connolly *et al.* (2018) and Connolly *et al.* (2015), the average remuneration rate used to estimate the costs of expanding eligibility for a GPVC was based on the average capitation and fee payments made to GPs in respect of existing GPVC holders (with adjustments to the capitation payment for the increases planned as part of the 2019 GP Agreement).

The approach to estimating public GP costs in the economic evaluation literature has varied. Brick *et al.* (2015) adopted the average GMS payment per eligible person as reported by the PCRS (i.e., total capitation and other fees/allowances divided by the number of eligible persons). It is important to note that there has since been a shift in how the average payment per eligible person is calculated by the PCRS. Prior to 2017, the numerator included payments to GPs for the miscellaneous schemes (i.e., Primary Childhood Immunisation Scheme, National Cancer Screening Service, etc.). From 2017 onwards, the numerator excludes these schemes. In an analysis of the cost of stroke in Ireland, Smith *et al.* (2012) estimated the average capitation payments per GP visit by age group (ranging from €13 to €78 per visit based on 2007/08 data) but did not include any fees or allowances.

For private GP prices, researchers have relied on available survey data, detailed below.

*Public GP visit cost – methods*

This analysis draws on the methods adopted in the existing literature and the public GP visit cost is estimated as follows.

In Equation 2, the average annual payment to GPs per person eligible under the MC/GPVC schemes is calculated.

$$\begin{array}{l} \text{Equation}\;2:\text{Average}\;\text{annual}\;\text{payment}\;\text{to}\;\text{GPs}\;\text{per}\;\text{eligible}\;\text{person}=\\ \;\frac{\mathrm{Total}\;\mathrm{payments}\;\mathrm{to}\;\mathrm{GPs}\;\mathrm{by}\;\mathrm{PCRS}\;(\mathrm{capitation},\;\mathrm{fees},\;\mathrm{allowances},\;\mathrm{etc})}{\mathrm{Number}\;\mathrm{of}\;\mathrm{eligible}\;\mathrm{persons}\;(\mathrm{MC}\;\&\;\mathrm{GPVC})} \end{array}$$

In Equation 3, the average annual number of GP visits per eligible person is estimated based on available survey data (detailed below). GP visiting rates vary by MC/GPVC status *i ^17^* and age group *j ^18^* and the number of eligible people is not even across these subgroups *ij*. The average annual number of GP visits in each subgroup (V _*ij*_) is multiplied by the proportion of the total MC/GPVC population in each subgroup (P _*ij*_) and these are summed to give an overall weighted average number of visits per cardholder.

$$\begin{array}{l} \text{Equation}\;3:\text{Weighted}\;\text{average}\;\text{annual}\;\text{number}\;\text{of}\;\text{visits}\;\text{per}\;\text{cardholder}=\\ \;\sum V_{ij}\;P_{ij} \end{array}$$

Equation 3: Weighted average annual number of visits per cardholder =

In Equation 4, the average annual payment per person is divided by the average annual number of GP visits per person to give the unit cost per public GP visit.

Equation 4: Unit cost per public GP visit =

$$\begin{array}{l} \text{Equation}\;4:\text{Unit}\;\text{cost}\;\text{per}\;\text{public}\;\text{GP}\;\text{visit}=\\ \;\frac{Average\;annual\;payment\;to\;GPs\;per\;eligible\;person\;(MC\;\&\;GPVC)}{Weighted\;average\;annual\;no.\;of\;visits\;per\;cardholder\;(MC\&\;GPVC)} \end{array}$$

There is an underlying assumption here that GP visits are all of equal length but further data are needed to examine variations in the duration of GP visits and the factors that drive these variations. Recent analysis by Pierse *et al.* (2019) demonstrate a useful method for collecting data on visit duration in Irish GP practices.

*Private GP visit cost – methods*

The most recent survey data available on private GP fees are identified.

**2.3.1.3 Data for public GP unit costs**

*Data on payments*

Data on payments to GPs are taken from the annual reports published by the PCRS (2016); PCRS (2017); PCRS (2018); PCRS (2019). Data on payments to GPs under the Maternity & Infant Care Scheme are available in the 2019 PCRS annual report.

To calculate Equation 1, in the baseline scenario (Scenario 1), total payments to GPs include almost all of the payments that are made by the PCRS to GPs in respect of MC and GPVC holders (including under 6s), see Table 9. The inclusion of the allowances for practice-based overhead supports, as well as the direct patient costs of capitation and fees, is consistent with the micro-costing PSSRU methodology. In line with Connolly *et al.* (2018), fees for asylum seekers and dispensing fees are excluded on the basis that these payments apply to specific practices and do not extend to the total MC/GPVC population.

**Table 9.** List of Payments to GPs by PCRS.

Payments to GPs: List of Items		Scenario 1 Baseline	Scenario 2 Sensitivity
Fees:			
	Capitation	✓	✓
	Special Claims/Services	✓	✓
	Out-of-Hours	✓	✓
	Dispensing	✗	✗
	Item of Service Contract	✗	✗
	Asylum Seekers	✗	✗
	Vaccinations	✓	✓
	Asthma Registration	✓	✓
	Asthma Capitation	✓	✓
	Contribution for GP Height Measure and Self Zeroing Scale	✓	✓
	Diabetes Capitation	✓	✓
	Diabetes Registration	✓	✓
Allowances:			
	Secretarial/Nursing	✓	✓
	Annual Leave	✓	✓
	Rostering/Out-of-Hours	✓	✓
	Medical Indemnity Insurance	✓	✓
	Rural Practice	✓	✓
	Study Leave	✓	✓
	Sick Leave	✓	✓
	Maternity Leave/Paternity Leave	✓	✓
	Locum and Practice Expenses	✓	✓
	Practice Development	✓	✓
Salaries:			
	Benefits to retired District Medical Officers and their dependents	✗	✗
	Former District Medical Officers	✗	✗
Superannuation Fund Contribution		✓	✓
Additional payments to GPs (Miscellaneous Schemes):			
	Primary Childhood Immunisation Scheme	✗	✓
	Health (Amendment) Act 1996	✗	✓
	Heartwatch	✗	✓
	National Cancer Screening Service	✗	✓
	Methadone/Opioid Substitution Treatment Scheme	✗	✓
Maternity & Infant Care Scheme		✓	✓

Note: GP = General Practitioner; PCRS = Primary Care Reimbursement Service

Payments to GPs under the Maternity & Infant Care Scheme are included, based on 2019 PCRS data ( PCRS, 2019). This is a universal scheme that applies to all expectant mothers, regardless of medical card status. For MC and GPVC holders, the payments to GPs under the Maternity & Infant Care Scheme are in addition to the PCRS capitation payments. Without further data on the medical card status of recipients of this Scheme, it is not possible to identify what proportion of the payments refers to MC and GPVC holders only.

Benefits to retired District Medical Officers are excluded on the basis that these payments do not reflect current costs of GP care. Salary payments to former DMOs are excluded based on the assumption that these services are additional to those provided by practice-based GPs.

Payments under the additional ‘Miscellaneous Schemes’ are excluded in the baseline scenario. The Primary Childhood Immunisation Scheme is assumed to cover services that are mainly provided by nurses within GP practices, and services provided within schools that are separate to practice-based GP services. Data indicate that a majority of GP practices (more than 80% by 2015) employ a Practice Nurse ( O'Kelly *et al.*, 2016). Payments under the Health (Amendment) Act 1996, Heartwatch, and Opioid Substitution Treatment Scheme are excluded on the basis that these apply to specific practices/recipients and do not extend to the total MC/GPVC population. Payments under the National Cancer Screening Service are excluded on the assumption that these payments cover screening visits for cervical cancer which are mainly undertaken by nurses rather than GPs.

For sensitivity, an alternative scenario is examined to reflect the difficulty in identifying what constitutes the total cost of a public GP visit, for example, the difficulty in separating out costs relating to services provided by a Practice Nurse. Scenario 2 is based on the baseline but includes the Miscellaneous Schemes given that some of the recipients are likely to be MC/GPVC holders, and to reflect the fact that not all GP practices employ a Practice Nurse.

*Data on numbers covered*

Data on the number of MC and GPVC holders, by age and sex, are published online on a monthly basis by the PCRS ^19^. The total number of cardholders are reported in the annual published PCRS reports, as at 31 ^st^ December in each year. The monthly online report that corresponds with the data included in each annual report is for January of the following year. For example, the total number of MC and GPVC holders as at 31 ^st^ December in 2019 as reported in the PCRS 2019 annual report was 2,068,868 (1,544,374 MC and 524,494 GPVC holders, PCRS, 2019) matching the data reported in the January 2020 monthly online PCRS report ^20^.

*Data on utilisation*

Data on GP utilisation for individuals aged 15+ years are available from the Healthy Ireland (HI) surveys for the years 2016, 2018 and 2019. For each of these years, data on the mean number of GP visits per annum by MC and GPVC status as well as by age group (16–44, 45–64, 65+ years) were received from Ipsos MRBI via the DOH.

Data on GP utilisation for children under the age of 16 years were included in the 2019 HI survey. The mean number of GP visits per annum for MC and GPVC holders by age group (0–5, 6–11, 12–15 years) was received from Ipsos MRBI via the DOH. The 2019 data on child utilisation rates are applied in the unit cost calculations for 2016, 2017 and 2018.

Key caveats to note about the GP utilisation data include:

- For GP utilisation rates aged 15 years and under, medical card status refers to that of the survey respondent and not the child. Medical card status of the child was not asked in the survey and thus there may be some mis-classification of child medical card status (e.g., where a child has a MC or GPVC but the adult survey respondent does not, or vice versa). GP visiting rates are on average 3–5 visits per annum for children aged 0–15 years in the 2019 survey. These rates are higher than earlier data on child GP utilisation rates (e.g., Wren *et al.*, 2015) but this is the case for other age groups too when comparing HI data with earlier data on GP use.
- For GP utilisation rates aged 15 years and under, sample sizes were too small to examine GP utilisation for MC and GPVC holders separately.
- GP visiting rates were not included in the 2017 HI survey and 2018 rates have been applied.
- There is potential underestimation of visits vis-à-vis the payments to GPs. The HI survey questions on GP utilisation specify that a range of visits should be excluded, such as visits to perform prescribed and scheduled procedures (e.g., injections) and this is potentially out of line with the range of allowances included in the payments to GPs under the baseline and sensitivity scenarios outlined in Table 9.

Using the HI data, Table 10 presents the weighted average annual number of GP visits for the MC/GPVC population from 2016—2019 (as per Equation 2). The average number of visits varies by almost 1 visit across the period indicating a high degree of variability in the underlying data from one year to the next. The Healthy Ireland questionnaire asks about GP use in the last 4 weeks. While this ensures more accurate recall, there is likely to be a margin of error when these visiting rates are extrapolated out to give an annual rate. To allow for a more stable picture of the average cost per public GP visit over time, the average number of GP visits over the time period 2016—2019 is calculated.

**Table 10.** Weighted average annual number of GP visits for MC/GPVC holders, 2016–2019.

	2016	2017	2018	2019	Average 2016—2019
Average number of GP visits per annum 2016—2019	6.35	5.47	5.48	6.34	5.91

Notes: GP = General Practitioner; MC = Medical Card; GPVC = GP Visit Card Sources: Healthy Ireland, 2016; Healthy Ireland, 2018; Healthy Ireland, 2019 MC & GPVC holders by age group: PCRS online portal https://www.sspcrs.ie/portal/annual-reporting [last accessed 08/12/2020]

**2.3.1.4 Comments on public GP unit costs**

*New GP Agreement*

It is important to note that the new 2019 GP Agreement ( DOH *et al.*, 2019) has implications for future estimates of public GP visit costs. The agreement includes plans for additional payments to GPs over a phased basis to support a range of measures to improve integrated healthcare delivery. The new 2019 GP Agreement has three main strands including: fee increases under the GMS contracts in return for delivery by GPs of a package of ‘Service Modernisation and Reform Measures’; introduction of a new ‘Integrated Model of Chronic Disease Prevention and Management’ and additional special items of service, supported by additional funding; and extension of eligibility for GP Visit Cards to all children of primary school age (under 13 years). The third strand, although included in the agreement, will be the subject of further negotiations between the DOH, HSE, and IMO.

The package of Service Modernisation and Reform Measures includes GP support for and cooperation with the implementation of community health networks, advances in eHealth and data management, medicines optimisation and other measures to improve contractual issues (e.g., GP practice profile, complaints procedures etc.). To support this package, the capitation fees (for ages 6+ years) have been set to increase in a phased basis over a 4-year period, 2019—2022, for GPs who sign up to the 2019 Agreement. This strand will see improvements in the delivery of primary care but does not in itself imply changes in visiting rates – i.e., payments increase, visits stay the same. Table 11 outlines the capitation fees for 2020 under the 2019 Agreement.

**Table 11.** Capitation payments to GPs, 2020.

	Capitation Payments, 2020 (€)									
	6-15		16-44		45-64		65-69		70+	
	Male	Female	Male	Female	Male	Female	Male	Female	At home	Private nursing home
Current capitation	43.29	43.79	55.26	90.37	110.38	121.29	116.28	129.72	271.62	434.15
Indicative Service Modernisation & Reform Fee	10.09	10.21	12.89	21.07	25.74	28.28	27.11	30.25	63.33	101.23
2020 Total	53.38	54.00	68.15	111.44	136.12	149.57	143.39	159.97	334.95	535.38

Note: GP = General Practitioner Source: DOH *et al.*, 2019

The Integrated Model of Chronic Disease Prevention and Management aims to provide a structured treatment programme for those with specified chronic diseases (including Diabetes Type 2, Asthma, Chronic Obstructive Pulmonary Disease, Cardiovascular Disease), as well as other measures to identify undiagnosed cases of chronic disease (or cases at high risk of Cardiovascular Disease or Diabetes), and provide annual preventive visits for patients identified with high risk of Cardiovascular Disease or Diabetes. Specific fees for the treatment, case finding, and preventive elements of the programme have been agreed and the programme will be rolled out over the period 2020–2023. This strand will see improvements in chronic disease prevention, diagnosis and management and implies changes in visiting rates (and possibly the duration of visits also), i.e., payments increase and visits increase.

The agreement also includes provisions for paying GPs for additional Special Items of Service. This includes payments for Therapeutic Phlebotomy for Haemochromatosis. Current admissions of MC and GPVC holders to acute/out-patient care for this treatment will be transferred to General Practice (approx. 3 visits per patient per year). GPs will also be paid for their role in handling involuntary admissions to acute mental health facilities, and for attending virtual consultations between GPs and Consultant Cardiologists to discuss patients with heart failure. This strand will see both increases in payments as well as visits.

*GP Practice Nurse*

Further analysis is also needed to examine the role of the Practice Nurse to avoid reporting duplicate/overlapping unit costs. A separate unit cost for a Practice Nurse is not included in this paper but it should be noted that some of the costs associated with Practice Nurse services are incorporated into the GP unit costs outlined above (e.g., allowances for nursing support, fees for services more likely to be provided by Practice Nurses). Further data on payments and roles and responsibilities within GP practices are required to estimate the unit cost of a GP Practice Nurse visit.

**2.3.1.5 Data for private GP unit costs**. Irish cost-of-illness studies and economic evaluations have relied on available survey data on private GP fees. An average private fee of €50 was applied in a number of studies in the period 2012—2014 (e.g., Connolly *et al.*, 2014; Gillespie *et al.*, 2013; Smith *et al.*, 2012). These studies drew on two independent price surveys: an ‘informal price check’ of 51 GPs in rural and urban locations by the former Competition Authority which found a price range of €45-60 in 2008 ( The Competition Authority, 2010: 10); and a survey by the former National Consumer Agency of 251 GPs which found an average price of €51 ( National Consumer Agency, 2010). In 2013, Brick *et al.* (2017) conducted a small telephone survey of 36 GP surgeries from three regions (South East, Midlands, Mid West) and found an average price of €47 per GP consultation in those regions.

The fifth wave of the HI survey (2018–2019) reported that 29% of adults paid up to €50 per GP visit and a further 24% paid up to €75 per visit ( Healthy Ireland, 2019). The average payment made by non-medical card holders in the fifth wave of the HI survey was €49.78 ^21^ indicating that an average private fee of €50 is still a reasonable estimate.

***2.3.2 Dentist unit costs***

**2.3.2.1 Structure of dental practice in Ireland**. Dental services in Ireland are delivered in a mixed public-private system and there are an estimated 44 dentists per 100,000 population ( DOH, 2019).

The majority of dental care is provided by private general dental practitioners and financed by out-of-pocket payments by individuals ( Nolan, 2019).

Publicly funded dental care is available through three different schemes: the Dental Treatment Services Scheme (DTSS), the Treatment Benefit Scheme (TBS) and the Public Dental Service (PDS) ( DOH, 2019; Nolan, 2019). The DTSS finances dental healthcare services for adult medical cardholders, the TBS subsidises a limited range of dental healthcare for non-medical cardholder adults with eligibility linked to their PRSI contributions, and the PDS finances dental care mainly for children of primary school age ( DOH, 2019; Nolan, 2019). The HSE reimburses private dental practitioners for services provided under the DTSS on a fee-for-service basis, and directly employs public sector dental practitioners to deliver the PDS. The TBS is administered separately by the Department of Employment Affairs and Social Protection ( Nolan, 2019).

**2.3.2.2 Data for public and private fees**. The schedule of fees payable to dentists under the DTSS is publicly available in the PCRS annual reports ( PCRS, 2016; PCRS, 2017; PCRS, 2018; PCRS, 2019) but it is important to note that these fees may not reflect actual costs.

There are limited survey estimates of the private fees charged by dentists with the average price for a dental consultation/examination close to €45 for the years 2010 to 2014 ( National Consumer Agency, 2010; WhatClinic.com, 2014). A more recent survey of private dental fees is underway and preliminary results for 2020 prices are reported here for the services of dental examination (excluding x-rays), cleaning (scale and polish by the dentist), and routine extraction. The survey covers a nationally representative sample of c. 100 private dentists in the Republic of Ireland ^22^.

***2.3.3 Long-term residential care unit costs***

**2.3.3.1 Structure of long-term residential care in Ireland**. Long-term residential care is delivered in a range of settings including private nursing homes, public facilities including extended care units, public nursing homes, and a small number of welfare homes, and in non-statutory/voluntary agencies.

The LTRC sector has been changing rapidly over recent years and approximately 75% of LTRC beds are now provided by private nursing homes (HIQA, 2014).

Although the majority of care takes place in private LTRC beds, the majority of residents are financed through the public Nursing Homes Support Scheme (NHSS), known as the ‘Fair Deal’ scheme ( Wren *et al.*, 2017). Applicants to the Fair Deal scheme receive a care needs assessment to confirm that long-term nursing home care is appropriate. The Fair Deal participant makes a contribution towards the cost of his/her care (based on an assessment of income and assets) and the HSE pays the balance ( Citizens Information Board, 2020).

**2.3.3.2 Data sources on costs**. Data are available on the costs and charges for care in the public and private LTRC facilities participating in the Fair Deal Scheme. These data are assumed to be representative of the LTRC sector given that in 2018, 94% of all LTRC facilities that were registered with HIQA participated in the Fair Deal Scheme ( C&AG, 2020) ^23^.

An individual weekly charge is calculated for each LTRC facility participating in the Fair Deal Scheme. The methods for calculating the weekly charge are different for public and private facilities.

Charges for public LTRC facilities are published by the HSE ^24^ and are based on costs of care including: pay (management, nursing and support staff) excluding superannuation costs, plus operating expenses to cover minor capital works, general equipment and furniture, training and education costs. However, it is important to note that the cost components and operating expense guidelines for calculating these weekly charge rates have not been updated since 2009 and this has been recently criticised ( C&AG, 2020).

The charges for private LTRC facilities are based on an agreed maximum price negotiated between each facility and the National Treatment Purchase Fund (NTPF) ^25^.

Given the differences in the methods, the data for public and private costs of LTRC are reported separately in this paper and it is noted that drawing meaningful comparisons between the public and private costs is difficult ( C&AG, 2020). For each year, there is a notable difference in the public and private costs with public costs being more than 50% higher than private costs over the period 2016—2020. The HSE has identified key factors contributing to these differences including higher nurse staffing ratios in public facilities than in private facilities, provision of LTRC services in remote locations that are less viable for private providers (e.g., Achill Island, Donegal), and others ( HSE, 2019c) but there has been no formal analysis of these cost drivers ( C&AG, 2020). A value-for-money review to examine the differences between public and private nursing home costs started in 2018 but has yet to be completed ^26^.

## 3 Unit cost tables

### 3.1 Micro-costed unit costs

***3.1.1 Overview.*** The tables in this section show the estimated unit costs for 11 publicly-employed, community-based, healthcare professionals calculated using micro-costing methods based on the PSSRU approach. These include allied health, public health nursing, and care support staff.

For each healthcare professional the data are presented as follows:

- BASELINE

○ Estimated unit costs for 2016–2019 at baseline overheads of 40%
○ Accompanying notes table (description and data sources)

- SENSITIVITY

○ LOW: Estimated unit costs for 2016–2019 at low overheads of 25%
○ HIGH: Estimated unit costs for 2016–2019 at high overheads of 60+%

While the baseline provides a useful mid-range estimate, there may be justification for applying the low/high sensitivity estimates depending on the focus of the study. For example, in a study where unit costs for a range support staff are being included, it may be reasonable to adjust the overheads downwards accordingly assuming that some of these support services are captured in the overhead costs and to avoid double counting. In these cases, transparency in reporting which unit cost is adopted is crucial to ensuring comparability across studies.

***3.1.2 Health and Social Care Professionals.*** A range of therapy and care professions are included in the Health & Social Care Professional (HSCP) staff category.

The following tables present unit costs for the professions that account for the largest proportion of HSCP professionals, measured in WTEs, employed by the HSE in community services in the years 2016–2019.

For example, in 2019, the HSE employed 8,357 WTE staff ^27^ in the HSCP category in community services ( HSE, 2019d). Therapy professions (3,298 WTEs), Social Care Workers (2,708 WTEs), Psychologists (912 WTEs) and Social Workers (818 WTEs) accounted for the largest proportion of these WTEs. Of the therapy professions: Occupational Therapy, Speech and Language Therapy, and Physiotherapy, accounted for the largest proportion of WTEs, with smaller numbers of Dietitians, and Podiatrists and Chiropodists ^28^.

Unit costs are presented in this paper for: Dietitian ( Tables 12a–c), Occupational Therapist ( Tables 13a–c), Physiotherapist ( Tables 14a–c), Podiatrist & Chiropodist ( Tables 15a–c), Psychologist ( Tables 16a–c), and Social Care Worker ( Tables 17a–c) ^29^

**3.1.2.1 Dietitian (DT)**

**Table 12a.** Estimated unit costs for a publicly employed, community based, Dietitian, 2016–2019.

BASELINE (OVERHEADS 40%)		2016	2017	2018	2019
Cost component		€	€	€	€
A	Wages/salary	54,578	55,328	56,274	57,025
B	Salary oncosts	16,441	16,028	15,748	16,585
C	Qualifications	-	-	-	-
D	Overheads	21,831	22,131	22,509	22,810
E	Capital overheads	9,413	9,458	7,565	7,732
F	Travel	-	-	-	-
G	Total costs (∑A–F)	102,263	102,945	102,096	104,153
Working time					
H	Number of hours worked per annum	1,601	1,595	1,599	1,598
I	Ratio of direct to indirect time	-	-	-	-
Unit costs		€	€	€	€
J	Unit cost per hour (G/H)	64	65	64	65

Cost component		Description	Sources
A	Wages/salary	Annual mean whole-time equivalent basic salary for Dietitian (DT), Senior. Senior DTs accounted for 69–72% of publicly employed DTs working in community-based services for the period 2016—2019.	HSE (2020) Health Service Personnel Census HSE, 2020a
B	Salary oncosts	Pay Related Social Insurance (PRSI) Contribution, calculated at 10.75–10.95% of annual mean WTE basic salary for DT, Senior for the period 2016—2019. Superannuation: weighted average of the public sector pension contribution rates for pre-2013 and post-2013 pension cohorts estimated by DPER (with adjustment for the Pension Related Deduction/Annual Superannuation Charge). Average pension contribution of 16–20% over the period 2016—2019 for publicly employed DTs.	DEASP, 2019, DEASP, 2020, DPER, 2017a DPER, 2017b Personal communication with IGEES, DOH, 04/01/2021 Personal communication with IGEES, DOH, 22/01/2021
C	Qualifications	Not included in this analysis	
D	Overheads	Estimated overhead costs for utilities (e.g., light, heat, telephone, internet), accommodation costs, office facilities, and general supplies as well as administrative and management staff costs using available guidelines. Baseline estimate of 40% of basic salary.	DOT, 2009 HIQA, 2018b HIQA, 2020 DPER, 2019 Curtis & Burns, 2019
E	Capital overheads	Estimated capital overhead costs based on average capital costs estimated by the Personal Social Services Research Unit. Estimate of 11–13% of total salary (basic plus oncosts) for the period 2016—2019.	Curtis & Burns, 2018; Curtis & Burns, 2019
F	Travel	Not included in this analysis	
H	Number of hours worked per annum	Total number of days worked per annum (number of working days in a given year minus annual leave entitlement and estimated sickness absence days) multiplied by number of hours worked per day.	Leave entitlement: HSE, 2009a; Personal communication with HSE, 04/02/2021 Absenteeism: HSE, 2017, HSE, 2018, HSE, 2019a HSE, 2019b Number of hours per week/day: Labour Relations Committee, 2013
I	Ratio of direct to indirect time	Not included in this analysis	

**Table 12b.** Estimated unit costs for publicly employed, community based, Dietitian, 2016–2019, SENSITIVITY LOW OVERHEADS.

LOW (OVERHEADS 25%)		2016	2017	2018	2019
Cost component		€	€	€	€
A	Wages/salary	54,578	55,328	56,274	57,025
B	Salary oncosts	16,441	16,028	15,748	16,585
C	Qualifications	-	-	-	-
D	Overheads	13,644	13,832	14,068	14,256
E	Capital overheads	9,413	9,458	7,565	7,732
F	Travel	-	-	-	-
G	Total costs (∑A–F)	94,076	94,646	93,655	95,599
Working time					
H	Number of hours worked per annum	1,601	1,595	1,599	1,598
I	Ratio of direct to indirect time	-	-	-	-
Unit costs		€	€	€	€
J	Unit cost per hour (G/H)	59	59	59	60

**Table 12c.** Estimated unit costs for publicly employed, community-based, Dietitian, 2016—2019, SENSITIVITY HIGH OVERHEADS.

HIGH (OVERHEADS 60+%)		2016	2017	2018	2019
Cost component		€	€	€	€
A	Wages/salary	54,578	55,328	56,274	57,025
B	Salary oncosts	16,441	16,028	15,748	16,585
C	Qualifications	-	-	-	-
D	Overheads	44,529	44,740	45,157	46,154
E	Capital overheads	9,413	9,458	7,565	7,732
F	Travel	-	-	-	-
G	Total costs (∑A–F)	124,961	125,554	124,744	127,497
Working time					
H	Number of hours worked per annum	1,601	1,595	1,599	1,598
I	Ratio of direct to indirect time	-	-	-	-
Unit costs		€	€	€	€
J	Unit cost per hour (G/H)	78	79	78	80

**3.1.2.2 Occupational Therapist (OT)**

**Table 13a.** Estimated unit costs for a publicly employed, community based, Occupational Therapist, 2016–2019.

BASELINE (OVERHEADS 40%)		2016	2017	2018	2019
Cost component		€	€	€	€
A	Wages/salary	54,578	55,328	56,274	57,025
B	Salary oncosts	16,441	16,028	15,748	16,585
C	Qualifications	-	-	-	-
D	Overheads	21,831	22,131	22,509	22,810
E	Capital overheads	9,413	9,458	7,565	7,732
F	Travel	-	-	-	-
G	Total costs (∑A–F)	102,263	102,945	102,096	104,153
Working time					
H	Number of hours worked per annum	1,601	1,595	1,599	1,598
I	Ratio of direct to indirect time	-	-	-	-
Unit costs		€	€	€	€
J	Unit cost per hour (G/H)	64	65	64	65

Cost component		Description	Sources
A	Wages/salary	Annual mean whole-time equivalent basic salary for Occupational Therapist (OT), Senior. Senior OTs accounted for 54-55% of publicly employed OTs working in community-based services for the period 2016—2019.	HSE (2020) Health Service Personnel Census HSE, 2020a
B	Salary oncosts	Pay Related Social Insurance (PRSI) Contribution, calculated at 10.75–-10.95% of annual mean WTE basic salary for OT, Senior for the period 2016—2019. Superannuation: weighted average of the public sector pension contribution rates for pre-2013 and post-2013 pension cohorts estimated by DPER (with adjustment for the Pension Related Deduction/ Annual Superannuation Charge). Average pension contribution of 16–20% over the period 2016—2019 for publicly employed OTs.	DEASP, 2019, DEASP, 2020 DPER, 2017a DPER, 2017b Personal communication with IGEES, DOH, 04/01/2021 Personal communication with IGEES, DOH, 22/01/2021
C	Qualifications	Not included in this analysis	
D	Overheads	Estimated overhead costs for utilities (e.g., light, heat, telephone, internet), accommodation costs, office facilities, and general supplies as well as administrative and management staff costs using available guidelines. Baseline estimate of 40% of basic salary.	DOT, 2009; HIQA, 2018b; HIQA, 2020; DPER, 2019; Curtis & Burns, 2019
E	Capital overheads	Estimated capital overhead costs based on average capital costs estimated by the Personal Social Services Research Unit. Estimate of 11–13% of total salary (basic plus oncosts) for the period 2016—2019.	Curtis & Burns, 2018; Curtis & Burns, 2019
F	Travel	Not included in this analysis	
H	Number of hours worked per annum	Total number of days worked per annum (number of working days in a given year minus annual leave entitlement and estimated sickness absence days) multiplied by number of hours worked per day.	Leave entitlement: HSE, 2009a; Personal communication with HSE, 04/02/2021 Absenteeism: HSE, 2017; HSE, 2018; HSE, 2019a HSE, 2019b Number of hours per week/day: Labour Relations Committee, 2013
I	Ratio of direct to indirect time	Not included in this analysis	

**Table 13b.** Estimated unit costs for publicly employed, community based, Occupational Therapist, 2016–2019, SENSITIVITY LOW OVERHEADS.

LOW (OVERHEADS 25%)		2016	2017	2018	2019
Cost component		€	€	€	€
A	Wages/salary	54,578	55,328	56,274	57,025
B	Salary oncosts	16,441	16,028	15,748	16,585
C	Qualifications	-	-	-	-
D	Overheads	13,644	13,832	14,068	14,256
E	Capital overheads	9,413	9,458	7,565	7,732
F	Travel	-	-	-	-
G	Total costs (∑A–F)	94,076	94,646	93,655	95,599
Working time					
H	Number of hours worked per annum	1,601	1,595	1,599	1,598
I	Ratio of direct to indirect time	-	-	-	-
Unit costs		€	€	€	€
J	Unit cost per hour (G/H)	59	59	59	60

**Table 13c.** Estimated unit costs for publicly employed, community-based, Occupational Therapist, 2016—2019, SENSITIVITY HIGH OVERHEADS.

HIGH (OVERHEADS 60+%)		2016	2017	2018	2019
Cost component		€	€	€	€
A	Wages/salary	54,578	55,328	56,274	57,025
B	Salary oncosts	16,441	16,028	15,748	16,585
C	Qualifications	-	-	-	-
D	Overheads	44,529	44,740	45,157	46,154
E	Capital overheads	9,413	9,458	7,565	7,732
F	Travel	-	-	-	-
G	Total costs (∑A–F)	124,961	125,554	124,744	127,497
Working time					
H	Number of hours worked per annum	1,601	1,595	1,599	1,598
I	Ratio of direct to indirect time	-	-	-	-
Unit costs		€	€	€	€
J	Unit cost per hour (G/H)	78	79	78	80

**3.1.2.3 Physiotherapist (PT)**

**Table 14a.** Estimated unit costs for a publicly employed, community based, Physiotherapist, 2016–2019.

BASELINE (OVERHEADS 40%)		2016	2017	2018	2019
Cost component		€	€	€	€
A	Wages/salary	54,578	55,328	56,274	57,025
B	Salary oncosts	16,441	16,028	15,748	16,585
C	Qualifications	-	-	-	-
D	Overheads	21,831	22,131	22,509	22,810
E	Capital overheads	9,413	9,458	7,565	7,732
F	Travel	-	-	-	-
G	Total costs (∑A–F)	102,263	102,945	102,096	104,153
Working time					
H	Number of hours worked per annum	1,601	1,595	1,599	1,598
I	Ratio of direct to indirect time	-	-	-	-
Unit costs		€	€	€	€
J	Unit cost per hour (G/H)	64	65	64	65

Cost component		Description	Sources
A	Wages/salary	Annual mean whole-time equivalent basic salary for Physiotherapist (PT), Senior. Senior PTs accounted for 62–64% of publicly employed PTs working in community-based services for the period 2016—2019.	HSE (2020) Health Service Personnel Census HSE, 2020a
B	Salary oncosts	Pay Related Social Insurance (PRSI) Contribution, calculated at 10.75– 10.95% of annual mean WTE basic salary for PT, Senior for the period 2016—2019. Superannuation: weighted average of the public sector pension contribution rates for pre-2013 and post-2013 pension cohorts estimated by DPER (with adjustment for the Pension Related Deduction/Annual Superannuation Charge). Average pension contribution of 16–20% over the period 2016—2019 for publicly employed PTs.	DEASP, 2019, DEASP, 2020 DPER, 2017a DPER, 2017b Personal communication with IGEES, DOH, 04/01/2021 Personal communication with IGEES, DOH, 22/01/2021
C	Qualifications	Not included in this analysis	
D	Overheads	Estimated overhead costs for utilities (e.g., light, heat, telephone, internet), accommodation costs, office facilities, and general supplies as well as administrative and management staff costs using available guidelines. Baseline estimate of 40% of basic salary.	DOT, 2009 HIQA, 2018b HIQA, 2020 DPER, 2019 Curtis & Burns, 2019
E	Capital overheads	Estimated capital overhead costs based on average capital costs estimated by the Personal Social Services Research Unit. Estimate of 11–13% of total salary (basic plus oncosts) for the period 2016—2019.	Curtis & Burns, 2018; Curtis & Burns, 2019
F	Travel	Not included in this analysis	
H	Number of hours worked per annum	Total number of days worked per annum (number of working days in a given year minus annual leave entitlement and estimated sickness absence days) multiplied by number of hours worked per day.	Leave entitlement: HSE, 2009a; Personal communication with HSE, 04/02/2021 Absenteeism: HSE, 2017, HSE, 2018, HSE, 2019a HSE, 2019b Number of hours per week/day: Labour Relations Committee, 2013
I	Ratio of direct to indirect time	Not included in this analysis	

**Table 14b.** Estimated unit costs for publicly employed, community based, Physiotherapist, 2016–2019, SENSITIVITY LOW OVERHEADS.

LOW (OVERHEADS 25%)		2016	2017	2018	2019
Cost component		€	€	€	€
A	Wages/salary	54,578	55,328	56,274	57,025
B	Salary oncosts	16,441	16,028	15,748	16,585
C	Qualifications	-	-	-	-
D	Overheads	13,644	13,832	14,068	14,256
E	Capital overheads	9,413	9,458	7,565	7,732
F	Travel	-	-	-	-
G	Total costs (∑A–F)	94,076	94,646	93,655	95,599
Working time					
H	Number of hours worked per annum	1,601	1,595	1,599	1,598
I	Ratio of direct to indirect time	-	-	-	-
Unit costs		€	€	€	€
J	Unit cost per hour (G/H)	59	59	59	60

**Table 14c.** Estimated unit costs for publicly employed, community-based, Physiotherapist, 2016—2019, SENSITIVITY HIGH OVERHEADS.

HIGH (OVERHEADS 60+%)		2016	2017	2018	2019
Cost component		€	€	€	€
A	Wages/salary	54,578	55,328	56,274	57,025
B	Salary oncosts	16,441	16,028	15,748	16,585
C	Qualifications	-	-	-	-
D	Overheads	44,529	44,740	45,157	46,154
E	Capital overheads	9,413	9,458	7,565	7,732
F	Travel	-	-	-	-
G	Total costs (∑A–F)	124,961	125,554	124,744	127,497
Working time					
H	Number of hours worked per annum	1,601	1,595	1,599	1,598
I	Ratio of direct to indirect time	-	-	-	-
Unit costs		€	€	€	€
J	Unit cost per hour (G/H)	78	79	78	80

**3.1.2.4 Podiatrist & Chiropodist (P&C)**

**Table 15a.** Estimated unit costs for a publicly employed, community based, Podiatrist/Chiropodist, 2016–2019.

BASELINE (OVERHEADS 40%)		2016	2017	2018	2019
Cost component		€	€	€	€
A	Wages/salary	54,578	55,328	56,274	57,025
B	Salary oncosts	16,441	16,028	15,748	16,585
C	Qualifications	-	-	-	-
D	Overheads	21,831	22,131	22,509	22,810
E	Capital overheads	9,413	9,458	7,565	7,732
F	Travel	-	-	-	-
G	Total costs (∑A–F)	102,263	102,945	102,096	104,153
Working time					
H	Number of hours worked per annum	1,601	1,595	1,599	1,598
I	Ratio of direct to indirect time	-	-	-	-
Unit costs		€	€	€	€
J	Unit cost per hour (G/H)	64	65	64	65

Cost component		Description	Sources
A	Wages/salary	Annual mean whole-time equivalent basic salary for Podiatrist/ Chiropodist (P&C), Senior. Senior P&Cs accounted for 70–81% of publicly employed P&Cs working in community-based services for the period 2016—2019.	HSE (2020) Health Service Personnel Census HSE, 2020a
B	Salary oncosts	Pay Related Social Insurance (PRSI) Contribution, calculated at 10.75– 10.95% of annual mean WTE basic salary for P&C, Senior for the period 2016—2019. Superannuation: weighted average of the public sector pension contribution rates for pre-2013 and post-2013 pension cohorts estimated by DPER (with adjustment for the Pension Related Deduction/Annual Superannuation Charge). Average pension contribution of 16–20% over the period 2016—2019 for publicly employed P&Cs.	DEASP, 2019, DEASP, 2020 DPER, 2017a DPER, 2017b Personal communication with IGEES, DOH, 04/01/2021 Personal communication with IGEES, DOH, 22/01/2021
C	Qualifications	Not included in this analysis	
D	Overheads	Estimated overhead costs for utilities (e.g., light, heat, telephone, internet), accommodation costs, office facilities, and general supplies as well as administrative and management staff costs using available guidelines. Baseline estimate of 40% of basic salary.	DOT, 2009 HIQA, 2018b HIQA, 2020 DPER, 2019 Curtis & Burns, 2019
E	Capital overheads	Estimated capital overhead costs based on average capital costs estimated by the Personal Social Services Research Unit. Estimate of 11–13% of total salary (basic plus oncosts) for the period 2016—2019.	Curtis & Burns, 2018; Curtis & Burns, 2019
F	Travel	Not included in this analysis	
H	Number of hours worked per annum	Total number of days worked per annum (number of working days in a given year minus annual leave entitlement and estimated sickness absence days) multiplied by number of hours worked per day.	Leave entitlement: HSE, 2009a; Personal communication with HSE, 04/02/2021 Absenteeism: HSE, 2017; HSE, 2018; HSE, 2019a HSE, 2019b Number of hours per week/day: Labour Relations Committee, 2013
I	Ratio of direct to indirect time	Not included in this analysis	

**Table 15b.** Estimated unit costs for publicly employed, community based, Podiatrist/Chiropodist, 2016–2019, SENSITIVITY LOW OVERHEADS.

LOW (OVERHEADS 25%)		2016	2017	2018	2019
Cost component		€	€	€	€
A	Wages/salary	54,578	55,328	56,274	57,025
B	Salary oncosts	16,441	16,028	15,748	16,585
C	Qualifications	-	-	-	-
D	Overheads	13,644	13,832	14,068	14,256
E	Capital overheads	9,413	9,458	7,565	7,732
F	Travel	-	-	-	-
G	Total costs (∑A–F)	94,076	94,646	93,655	95,599
Working time					
H	Number of hours worked per annum	1,601	1,595	1,599	1,598
I	Ratio of direct to indirect time	-	-	-	-
Unit costs		€	€	€	€
J	Unit cost per hour (G/H)	59	59	59	60

**Table 15c.** Estimated unit costs for publicly employed, community-based, Podiatrist/Chiropodist, 2016—2019, SENSITIVITY HIGH OVERHEADS.

HIGH (OVERHEADS 60+%)		2016	2017	2018	2019
Cost component		€	€	€	€
A	Wages/salary	54,578	55,328	56,274	57,025
B	Salary oncosts	16,441	16,028	15,748	16,585
C	Qualifications	-	-	-	-
D	Overheads	44,529	44,740	45,157	46,154
E	Capital overheads	9,413	9,458	7,565	7,732
F	Travel	-	-	-	-
G	Total costs (∑A–F)	124,961	125,554	124,744	127,497
Working time					
H	Number of hours worked per annum	1,601	1,595	1,599	1,598
I	Ratio of direct to indirect time	-	-	-	-
Unit costs		€	€	€	€
J	Unit cost per hour (G/H)	78	79	78	80

**3.1.2.5 Psychologist (PSY)**

**Table 16a.** Estimated unit costs for a publicly employed, community based, Psychologist, 2016–2019.

BASELINE (OVERHEADS 40%)		2016	2017	2018	2019
Cost component		€	€	€	€
A	Wages/salary	79,895	81,685	85,729	86,874
B	Salary oncosts	24,068	23,663	23,991	25,266
C	Qualifications	-	-	-	-
D	Overheads	31,958	32,674	34,292	34,750
E	Capital overheads	13,780	13,964	11,525	11,779
F	Travel	-	-	-	-
G	Total costs (∑A–F)	149,700	151,986	155,536	158,669
Working time					
H	Number of hours worked per annum	1,601	1,595	1,599	1,598
I	Ratio of direct to indirect time	-	-	-	-
Unit costs		€	€	€	€
J	Unit cost per hour (G/H)	94	95	97	99

Cost component		Description	Sources
A	Wages/salary	Annual mean whole-time equivalent basic salary for Psychologist (PSY), Senior. Senior PSYs accounted for 40–43% of publicly employed PSYs working in community-based services for the period 2016—2019. (No one grade accounts for more than 50% of WTE PSYs in the community- based services, but the Senior grade accounts for the largest share).	HSE (2020) Health Service Personnel Census HSE, 2020a
B	Salary oncosts	Pay Related Social Insurance (PRSI) Contribution, calculated at 10.75– 10.95% of annual mean WTE basic salary for PSY, Senior for the period 2016—2019. Superannuation: weighted average of the public sector pension contribution rates for pre-2013 and post-2013 pension cohorts estimated by DPER (with adjustment for the Pension Related Deduction/Annual Superannuation Charge). Average pension contribution of 16–20% over the period 2016—2019 for publicly employed PSYs.	DEASP, 2019, DEASP, 2020 DPER, 2017a DPER, 2017b Personal communication with IGEES, DOH, 04/01/2021 Personal communication with IGEES, DOH, 22/01/2021
C	Qualifications	Not included in this analysis	
D	Overheads	Estimated overhead costs for utilities (e.g., light, heat, telephone, internet), accommodation costs, office facilities, and general supplies as well as administrative and management staff costs using available guidelines. Baseline estimate of 40% of basic salary.	DOT, 2009 HIQA, 2018b HIQA, 2020 DPER, 2019 Curtis & Burns, 2019
E	Capital overheads	Estimated capital overhead costs based on average capital costs estimated by the Personal Social Services Research Unit. Estimate of 11–13% of total salary (basic plus oncosts) for the period 2016—2019.	Curtis & Burns, 2018; Curtis & Burns, 2019
F	Travel	Not included in this analysis	
H	Number of hours worked per annum	Total number of days worked per annum (number of working days in a given year minus annual leave entitlement and estimated sickness absence days) multiplied by number of hours worked per day.	Leave entitlement: HSE, 2009a; Personal communication with HSE, 04/02/2021 Absenteeism: HSE, 2017; HSE, 2018; HSE, 2019a HSE, 2019b Number of hours per week/day: Labour Relations Committee, 2013
I	Ratio of direct to indirect time	Not included in this analysis	

**Table 16b.** Estimated unit costs for publicly employed, community based, Psychologist, 2016–2019, SENSITIVITY LOW OVERHEADS.

LOW (OVERHEADS 25%)		2016	2017	2018	2019
Cost component		€	€	€	€
A	Wages/salary	79,895	81,685	85,729	86,874
B	Salary oncosts	24,068	23,663	23,991	25,266
C	Qualifications	-	-	-	-
D	Overheads	19,974	20,421	21,432	21,718
E	Capital overheads	13,780	13,964	11,525	11,779
F	Travel	-	-	-	-
G	Total costs (∑A–F)	137,716	139,733	142,677	145,638
Working time					
H	Number of hours worked per annum	1,601	1,595	1,599	1,598
I	Ratio of direct to indirect time	-	-	-	-
Unit costs		€	€	€	€
J	Unit cost per hour (G/H)	86	88	89	91

**Table 16c.** Estimated unit costs for publicly employed, community-based, Psychologist, 2016—2019, SENSITIVITY HIGH OVERHEADS.

HIGH (OVERHEADS 60+%)		2016	2017	2018	2019
Cost component		€	€	€	€
A	Wages/salary	79,895	81,685	85,729	86,874
B	Salary oncosts	24,068	23,663	23,991	25,266
C	Qualifications	-	-	-	-
D	Overheads	65,184	66,053	68,794	70,312
E	Capital overheads	13,780	13,964	11,525	11,779
F	Travel	-	-	-	-
G	Total costs (∑A–F)	182,927	185,365	190,039	194,232
Working time					
H	Number of hours worked per annum	1,601	1,595	1,599	1,598
I	Ratio of direct to indirect time	-	-	-	-
Unit costs		€	€	€	€
J	Unit cost per hour (G/H)	114	116	119	122

**3.1.2.6 Social Care Worker (SCW)**

**Table 17a.** Estimated unit costs for a publicly employed, community based, Social Care Worker, 2016–2019.

BASELINE (OVERHEADS 40%)		2016	2017	2018	2019
Cost component		€	€	€	€
A	Wages/salary	37,663	38,413	39,147	39,670
B	Salary oncosts	11,346	11,128	10,955	11,538
C	Qualifications	-	-	-	-
D	Overheads	15,065	15,365	15,659	15,868
E	Capital overheads	6,496	6,566	5,263	5,379
F	Travel	-	-	-	-
G	Total costs (∑A–F)	70,569	71,472	71,023	72,454
Working time					
H	Number of hours worked per annum	1,636	1,631	1,635	1,634
I	Ratio of direct to indirect time	-	-	-	-
Unit costs		€	€	€	€
J	Unit cost per hour (G/H)	43	44	43	44

Cost component		Description	Sources
A	Wages/salary	Annual mean whole-time equivalent basic salary for Social Care Worker (SCW), basic grade. SCWs (basic grade) accounted for 82% of publicly employed SCWs working in community-based services for the period 2016—2019.	HSE, (2020) Health Service Personnel Census HSE, 2020a
B	Salary oncosts	Pay Related Social Insurance (PRSI) Contribution, calculated at 10.75– 10.95% of annual mean WTE basic salary for SCW (basic grade) for the period 2016—2019. Superannuation: weighted average of the public sector pension contribution rates for pre-2013 and post-2013 pension cohorts estimated by DPER (with adjustment for the Pension Related Deduction/Annual Superannuation Charge). Average pension contribution of 16–20% over the period 2016—2019 for publicly employed SCWs.	DEASP, 2019, DEASP, 2020 DPER, 2017a DPER, 2017b Personal communication with IGEES, DOH, 04/01/2021 Personal communication with IGEES, DOH, 22/01/2021
C	Qualifications	Not included in this analysis	
D	Overheads	Estimated overhead costs for utilities (e.g., light, heat, telephone, internet), accommodation costs, office facilities, and general supplies as well as administrative and management staff costs using available guidelines. Baseline estimate of 40% of basic salary.	DOT, 2009 HIQA, 2018b HIQA, 2020 DPER, 2019 Curtis & Burns, 2019
E	Capital overheads	Estimated capital overhead costs based on average capital costs estimated by the Personal Social Services Research Unit. Estimate of 11–13% of total salary (basic plus oncosts) for the period 2016—2019.	Curtis & Burns, 2018; Curtis & Burns, 2019
F	Travel	Not included in this analysis	
H	Number of hours worked per annum	Total number of days worked per annum (number of working days in a given year minus annual leave entitlement and estimated sickness absence days) multiplied by number of hours worked per day.	Leave entitlement: HSE, 2009a; Personal communication with HSE, 04/02/2021 Absenteeism: HSE, 2017; HSE, 2018; HSE, 2019a HSE, 2019b Number of hours per week/day: Labour Relations Committee, 2013
I	Ratio of direct to indirect time	Not included in this analysis	

**Table 17b.** Estimated unit costs for publicly employed, community based, Social Care Worker, 2016–2019, SENSITIVITY LOW OVERHEADS.

LOW (OVERHEADS 25%)		2016	2017	2018	2019
Cost component		€	€	€	€
A	Wages/salary	37,663	38,413	39,147	39,670
B	Salary oncosts	11,346	11,128	10,955	11,538
C	Qualifications	-	-	-	-
D	Overheads	9,416	9,603	9,787	9,917
E	Capital overheads	6,496	6,566	5,263	5,379
F	Travel	-	-	-	-
G	Total costs (∑A–F)	64,920	65,710	65,151	66,504
Working time					
H	Number of hours worked per annum	1,636	1,631	1,635	1,634
I	Ratio of direct to indirect time	-	-	-	-
Unit costs		€	€	€	€
J	Unit cost per hour (G/H)	40	40	40	41

**Table 17c.** Estimated unit costs for publicly employed, community based, Social Care Worker, 2016—2019, SENSITIVITY HIGH OVERHEADS.

HIGH (OVERHEADS 60+%)		2016	2017	2018	2019
Cost component		€	€	€	€
A	Wages/salary	37,663	38,413	39,147	39,670
B	Salary oncosts	11,346	11,128	10,955	11,538
C	Qualifications	-	-	-	-
D	Overheads	30,728	31,062	31,414	32,107
E	Capital overheads	6,496	6,566	5,263	5,379
F	Travel	-	-	-	-
G	Total costs (∑A–F)	86,232	87,168	86,778	88,693
Working time					
H	Number of hours worked per annum	1,636	1,631	1,635	1,634
I	Ratio of direct to indirect time	-	-	-	-
Unit costs		€	€	€	€
J	Unit cost per hour (G/H)	53	53	53	54

***3.1.3 Nursing and Midwifery.*** The HSE employed more than 14,800 WTE staff in the Nursing & Midwifery category in community services in 2019 ( HSE, 2019d) ^30^.

Public Health Nurses account for more than 1,500 WTEs within this category and the unit cost is presented below ( Tables 18a–c). Other nursing cadres that account for a large proportion of WTEs in this category include Staff Nurses (e.g., Psychiatric, Intellectual Disability, General/Children’s) ^31^.

**3.1.3.1 Public Health Nurse (PHN)**

**Table 18a.** Estimated unit costs for a publicly employed, community based, Public Health Nurse, 2016–2019.

BASELINE (OVERHEADS 40%)		2016	2017	2018	2019
Cost component		€	€	€	€
A	Wages/salary	49,544	50,294	51,177	51,861
B	Salary oncosts	14,429	14,067	13,810	14,564
C	Qualifications	-	-	-	-
D	Overheads	19,818	20,118	20,471	20,744
E	Capital overheads	6,380	6,419	6,372	6,513
F	Travel	-	-	-	-
G	Total costs (∑A–F)	90,171	90,897	91,829	93,682
Working time					
H	Number of hours worked per annum	1,669	1,663	1,669	1,668
I	Ratio of direct to indirect time	-	-	-	-
Unit costs		€	€	€	€
J	Unit cost per hour (G/H)	54	55	55	56

Cost component		Description	Sources
A	Wages/salary	Annual mean whole-time equivalent basic salary for Public Health Nurse (PHN).	HSE (2020) Health Service Personnel Census HSE, 2020a
B	Salary oncosts	Pay Related Social Insurance (PRSI) Contribution, calculated at 10.75–10.95% of annual mean WTE basic salary for PHN for the period 2016—2019. Superannuation: weighted average of the public sector pension contribution rates for pre-2013 and post-2013 pension cohorts estimated by DPER (with adjustment for the Pension Related Deduction/Annual Superannuation Charge). Average pension contribution of 15–19% over the period 2016—2019 for publicly employed PHNs.	DEASP, 2019, DEASP, 2020 DPER, 2017a DPER, 2017b Personal communication with IGEES, DOH, 04/01/2021 Personal communication with IGEES, DOH, 22/01/2021
C	Qualifications	Not included in this analysis	
D	Overheads	Estimated overhead costs for utilities (e.g., light, heat, telephone, internet), accommodation costs, office facilities, and general supplies as well as administrative and management staff costs using available guidelines. Baseline estimate of 40% of basic salary.	DOT, 2009 HIQA, 2018b HIQA, 2020 DPER, 2019 Curtis & Burns, 2019
E	Capital overheads	Estimated capital overhead costs based on average capital costs estimated by the Personal Social Services Research Unit. Estimate of 10% of total salary (basic plus oncosts) for the period 2016—2019.	Curtis & Burns, 2018; Curtis & Burns, 2019
F	Travel	Not included in this analysis	
H	Number of hours worked per annum	Total number of days worked per annum (number of working days in a given year minus annual leave entitlement and estimated sickness absence days) multiplied by number of hours worked per day.	Leave entitlement: HSE, 2009a; Personal communication with HSE, 04/02/2021 Absenteeism: HSE, 2017; HSE, 2018; HSE, 2019a HSE, 2019b Number of hours per week/day: Labour Relations Committee, 2013
I	Ratio of direct to indirect time	Not included in this analysis	

**Table 18b.** Estimated unit costs for publicly employed, community based, Public Health Nurse, 2016–2019, SENSITIVITY LOW OVERHEADS.

LOW (OVERHEADS 25%)		2016	2017	2018	2019
Cost component		€	€	€	€
A	Wages/salary	49,544	50,294	51,177	51,861
B	Salary oncosts	14,429	14,067	13,810	14,564
C	Qualifications	-	-	-	-
D	Overheads	12,386	12,574	12,794	12,965
E	Capital overheads	6,380	6,419	6,372	6,513
F	Travel	-	-	-	-
G	Total costs (∑A–F)	82,739	83,353	84,153	85,903
Working time					
H	Number of hours worked per annum	1,669	1,663	1,669	1,668
I	Ratio of direct to indirect time	-	-	-	-
Unit costs		€	€	€	€
J	Unit cost per hour (G/H)	50	50	50	52

**Table 18c.** Estimated unit costs for publicly employed, community-based, Public Health Nurse, 2016—2019, SENSITIVITY HIGH OVERHEADS.

HIGH (OVERHEADS 60+%)		2016	2017	2018	2019
Cost component		€	€	€	€
A	Wages/salary	49,544	50,294	51,177	51,861
B	Salary oncosts	14,429	14,067	13,810	14,564
C	Qualifications	-	-	-	-
D	Overheads	40,111	40,354	40,747	41,649
E	Capital overheads	6,380	6,419	6,372	6,513
F	Travel	-	-	-	-
G	Total costs (∑A–F)	110,464	111,134	112,105	114,587
Working time					
H	Number of hours worked per annum	1,654	1,648	1,654	1,653
I	Ratio of direct to indirect time	-	-	-	-
Unit costs		€	€	€	€
J	Unit cost per hour (G/H)	67	67	68	69

***3.1.4 Patient and Client Care***

**3.1.4.1 Overview of Patient & Client Care**. The HSE employs more than 18,000 WTE staff in the Patient & Client Care staff category and within that category, the majority are ‘Health Care Assistants’ of various types (12,182 WTEs in December, 2019), and Health Care Support Assistants, formerly called Home Helps (3,569 WTEs in December, 2019) ( HSE, 2019d).

Specifically, Attendants (Multi-Task), Care Assistants (Disability Services), and Health Care Assistants make up the majority of the WTEs in the wider ‘Health Care Assistant’ staff group. The unit costs for these ( Table 19– Table 21) and for Health Care Support Assistants ( Tables 22a-c) are presented below.

**3.1.4.2 Attendant, Multi-Task (ATT)**

**Table 19a.** Estimated unit costs for a publicly employed, community based, Attendant (Multi-Task), 2016–2019.

BASELINE (OVERHEADS 40%)		2016	2017	2018	2019
Cost component		€	€	€	€
A	Wages/salary	30,107	30,857	31,497	31,917
B	Salary oncosts	8,768	8,630	8,499	8,964
C	Qualifications	-	-	-	-
D	Overheads	12,043	12,343	12,599	12,767
E	Capital overheads	5,153	5,234	4,201	4,294
F	Travel	-	-	-	-
G	Total costs (∑A–F)	56,071	57,064	56,795	57,942
Working time					
H	Number of hours worked per annum	1,689	1,679	1,683	1,684
I	Ratio of direct to indirect time	-	-	-	-
Unit costs		€	€	€	€
J	Unit cost per hour (G/H)	33	34	34	34

Cost component		Description	Sources
A	Wages/salary	Annual mean whole-time equivalent basic salary for Attendant, Multi-Task (ATT).	HSE (2020) Health Service Personnel Census HSE, 2020a
B	Salary oncosts	Pay Related Social Insurance (PRSI) Contribution, calculated at 10.75–10.95% of annual mean WTE basic salary for ATT for the period 2016—2019. Superannuation: weighted average of the public sector pension contribution rates for pre-2013 and post-2013 pension cohorts estimated by DPER (with adjustment for the Pension Related Deduction/Annual Superannuation Charge). Average pension contribution of 15–19% over the period 2016—2019 for publicly employed ATTs.	DEASP, 2019, DEASP 2020 DPER, 2017a DPER, 2017b Personal communication with IGEES, DOH, 04/01/2021 Personal communication with IGEES, DOH, 22/01/2021
C	Qualifications	Not included in this analysis	
D	Overheads	Estimated overhead costs for utilities (e.g., light, heat, telephone, internet), accommodation costs, office facilities, and general supplies as well as administrative and management staff costs using available guidelines. Baseline estimate of 40% of basic salary.	DOT, 2009 HIQA, 2018b HIQA, 2020 DPER, 2019 Curtis & Burns, 2019
E	Capital overheads	Estimated capital overhead costs based on average capital costs estimated by the Personal Social Services Research Unit. Estimate of 11–13% of total salary (basic plus oncosts) for the period 2016—2019.	Curtis & Burns, 2018; Curtis & Burns, 2019
F	Travel	Not included in this analysis	
H	Number of hours worked per annum	Total number of days worked per annum (number of working days in a given year minus annual leave entitlement and estimated sickness absence days) multiplied by number of hours worked per day.	Leave entitlement: HSE, 2009a; Personal communication with HSE, 04/02/2021 Absenteeism: HSE, 2017; HSE, 2018; HSE, 2019a HSE, 2019b Number of hours per week/day: Labour Relations Committee, 2013
I	Ratio of direct to indirect time	Not included in this analysis	

**Table 19b.** Estimated unit costs for publicly employed, community based, Attendant (Multi-Task), 2016–2019, SENSITIVITY LOW OVERHEADS.

LOW (OVERHEADS 25%)		2016	2017	2018	2019
Cost component		€	€	€	€
A	Wages/salary	30,107	30,857	31,497	31,917
B	Salary oncosts	8,768	8,630	8,499	8,964
C	Qualifications	-	-	-	-
D	Overheads	7,527	7,714	7,874	7,979
E	Capital overheads	5,153	5,234	4,201	4,294
F	Travel	-	-	-	-
G	Total costs (∑A–F)	51,555	52,436	52,071	53,154
Working time					
H	Number of hours worked per annum	1,689	1,679	1,683	1,684
I	Ratio of direct to indirect time	-	-	-	-
Unit costs		€	€	€	€
J	Unit cost per hour (G/H)	31	31	31	32

**Table 19c.** Estimated unit costs for publicly employed, community-based, Attendant (Multi-Task), 2016—2019, SENSITIVITY HIGH OVERHEADS.

HIGH (OVERHEADS 60+%)		2016	2017	2018	2019
Cost component		€	€	€	€
A	Wages/salary	30,107	30,857	31,497	31,917
B	Salary oncosts	8,768	8,630	8,499	8,964
C	Qualifications	-	-	-	-
D	Overheads	24,375	24,759	25,077	25,632
E	Capital overheads	5,153	5,234	4,201	4,294
F	Travel	-	-	-	-
G	Total costs (∑A–F)	68,403	69,480	69,274	70,807
Working time					
H	Number of hours worked per annum	1,689	1,679	1,683	1,684
I	Ratio of direct to indirect time	-	-	-	-
Unit costs		€	€	€	€
J	Unit cost per hour (G/H)	40	41	41	42

**3.1.4.3 Care Assistant, Disability (CAD)**

**Table 21a.** Estimated unit costs for a publicly employed, community based, Health Care Assistant, 2016–2019.

BASELINE (OVERHEADS 40%)		2016	2017	2018	2019
Cost component		€	€	€	€
A	Wages/salary	30,107	30,857	31,497	31,917
B	Salary oncosts	8,768	8,630	8,499	8,964
C	Qualifications	-	-	-	-
D	Overheads	12,043	12,343	12,599	12,767
E	Capital overheads	5,153	5,234	4,201	4,294
F	Travel	-	-	-	-
G	Total costs (∑A–F)	56,071	57,064	56,795	57,942
Working time					
H	Number of hours worked per annum	1,689	1,679	1,683	1,684
I	Ratio of direct to indirect time	-	-	-	-
Unit costs		€	€	€	€
J	Unit cost per hour (G/H)	33	34	34	34

Cost component		Description	Sources
A	Wages/salary	Annual mean whole-time equivalent basic salary for Health Care Assistant (HCA).	HSE (2020) Health Service Personnel Census HSE, 2020a
B	Salary oncosts	Pay Related Social Insurance (PRSI) Contribution, calculated at 10.75–10.95% of annual mean WTE basic salary for HCA for the period 2016—2019. Superannuation: weighted average of the public sector pension contribution rates for pre-2013 and post-2013 pension cohorts estimated by DPER (with adjustment for the Pension Related Deduction/ Annual Superannuation Charge). Average pension contribution of 15–19% over the period 2016—2019 for publicly employed HCAs.	DEASP, 2019, DEASP, 2020 DPER, 2017a DPER, 2017b Personal communication with IGEES, DOH, 04/01/2021 Personal communication with IGEES, DOH, 22/01/2021
C	Qualifications	Not included in this analysis	
D	Overheads	Estimated overhead costs for utilities (e.g., light, heat, telephone, internet), accommodation costs, office facilities, and general supplies as well as administrative and management staff costs using available guidelines. Baseline estimate of 40% of basic salary.	DOT, 2009 HIQA, 2018b HIQA, 2020 DPER, 2019 Curtis & Burns, 2019
E	Capital overheads	Estimated capital overhead costs based on average capital costs estimated by the Personal Social Services Research Unit. Estimate of 11–13% of total salary (basic plus oncosts) for the period 2016—2019.	Curtis & Burns, 2018; Curtis & Burns, 2019
F	Travel	Not included in this analysis	
H	Number of hours worked per annum	Total number of days worked per annum (number of working days in a given year minus annual leave entitlement and estimated sickness absence days) multiplied by number of hours worked per day.	Leave entitlement: HSE, 2009a; Personal communication with HSE, 04/02/2021 Absenteeism: HSE, 2017; HSE, 2018; HSE, 2019a HSE, 2019b Number of hours per week/day: Labour Relations Committee, 2013
I	Ratio of direct to indirect time	Not included in this analysis	

**Table 21b.** Estimated unit costs for publicly employed, community based, Health Care Assistant, 2016–2019, SENSITIVITY LOW OVERHEADS.

LOW (OVERHEADS 25%)		2016	2017	2018	2019
Cost component		€	€	€	€
A	Wages/salary	30,107	30,857	31,497	31,917
B	Salary oncosts	8,768	8,630	8,499	8,964
C	Qualifications	-	-	-	-
D	Overheads	7,527	7,714	7,874	7,979
E	Capital overheads	5,153	5,234	4,201	4,294
F	Travel	-	-	-	-
G	Total costs (∑A–F)	51,555	52,436	52,071	53,154
Working time					
H	Number of hours worked per annum	1,689	1,679	1,683	1,684
I	Ratio of direct to indirect time	-	-	-	-
Unit costs		€	€	€	€
J	Unit cost per hour (G/H)	31	31	31	32

**Table 21c.** Estimated unit costs for publicly employed, community-based, Dietitian, 2016—2019, SENSITIVITY HIGH OVERHEADS.

HIGH (OVERHEADS 60+%)		2016	2017	2018	2019
Cost component		€	€	€	€
A	Wages/salary	30,107	30,857	31,497	31,917
B	Salary oncosts	8,768	8,630	8,499	8,964
C	Qualifications	-	-	-	-
D	Overheads	24,375	24,759	25,077	25,632
E	Capital overheads	5,153	5,234	4,201	4,294
F	Travel	-	-	-	-
G	Total costs (∑A–F)	68,403	69,480	69,274	70,807
Working time					
H	Number of hours worked per annum	1,689	1,679	1,683	1,684
I	Ratio of direct to indirect time	-	-	-	-
Unit costs		€	€	€	€
J	Unit cost per hour (G/H)	40	41	41	42

**3.1.4.4 Health Care Assistant (HCA)**

**Table 20a.** Estimated unit costs for a publicly employed, community based, Care Assistant (Disability), 2016–2019.

BASELINE (OVERHEADS 40%)		2016	2017	2018	2019
Cost component		€	€	€	€
A	Wages/salary	31,409	32,159	32,815	33,253
B	Salary oncosts	9,148	8,995	8,855	9,339
C	Qualifications	-	-	-	-
D	Overheads	12,564	12,864	13,126	13,301
E	Capital overheads	5,376	5,455	4,377	4,474
F	Travel	-	-	-	-
G	Total costs (∑A–F)	58,496	59,472	59,173	60,368
Working time					
H	Number of hours worked per annum	1,689	1,679	1,683	1,684
I	Ratio of direct to indirect time	-	-	-	-
Unit costs		€	€	€	€
J	Unit cost per hour (G/H)	35	35	35	36

Cost component		Description	Sources
A	Wages/salary	Annual mean whole-time equivalent basic salary for Care Assistant (Disability) (CAD).	HSE (2020) Health Service Personnel Census HSE, 2020a
B	Salary oncosts	Pay Related Social Insurance (PRSI) Contribution, calculated at 10.75– 10.95% of annual mean WTE basic salary for CAD for the period 2016—2019. Superannuation: weighted average of the public sector pension contribution rates for pre-2013 and post-2013 pension cohorts estimated by DPER (with adjustment for the Pension Related Deduction/ Annual Superannuation Charge). Average pension contribution of 15– 19% over the period 2016—2019 for publicly employed CADs.	DEASP, 2019, DEASP, 2020 DPER, 2017a DPER, 2017b Personal communication with IGEES, DOH, 04/01/2021 Personal communication with IGEES, DOH, 22/01/2021
C	Qualifications	Not included in this analysis	
D	Overheads	Estimated overhead costs for utilities (e.g., light, heat, telephone, internet), accommodation costs, office facilities, and general supplies as well as administrative and management staff costs using available guidelines. Baseline estimate of 40% of basic salary.	DOT, 2009 HIQA, 2018b HIQA, 2020 DPER, 2019 Curtis & Burns, 2019
E	Capital overheads	Estimated capital overhead costs based on average capital costs estimated by the Personal Social Services Research Unit. Estimate of 11–13% of total salary (basic plus oncosts) for the period 2016—2019.	Curtis & Burns, 2018; Curtis & Burns, 2019
F	Travel	Not included in this analysis	
H	Number of hours worked per annum	Total number of days worked per annum (number of working days in a given year minus annual leave entitlement and estimated sickness absence days) multiplied by number of hours worked per day.	Leave entitlement: HSE, 2009a; Personal communication with HSE, 04/02/2021 Absenteeism: HSE, 2017; HSE, 2018; HSE, 2019a HSE, 2019b Number of hours per week/day: Labour Relations Committee, 2013
I	Ratio of direct to indirect time	Not included in this analysis	

**Table 20b.** Estimated unit costs for publicly employed, community based, Care Assistant (Disability), 2016–2019, SENSITIVITY LOW OVERHEADS.

LOW (OVERHEADS 25%)		2016	2017	2018	2019
Cost component		€	€	€	€
A	Wages/salary	31,409	32,159	32,815	33,253
B	Salary oncosts	9,148	8,995	8,855	9,339
C	Qualifications	-	-	-	-
D	Overheads	7,852	8,040	8,204	8,313
E	Capital overheads	5,376	5,455	4,377	4,474
F	Travel	-	-	-	-
G	Total costs (∑A–F)	53,785	54,648	54,251	55,380
Working time					
H	Number of hours worked per annum	1,689	1,679	1,683	1,684
I	Ratio of direct to indirect time	-	-	-	-
Unit costs		€	€	€	€
J	Unit cost per hour (G/H)	32	33	32	33

**Table 20c.** Estimated unit costs for publicly employed, community based, Care Assistant (Disability), 2016—2019, SENSITIVITY HIGH OVERHEADS.

HIGH (OVERHEADS 60+%)		2016	2017	2018	2019
Cost component		€	€	€	€
A	Wages/salary	31,409	32,159	32,815	33,253
B	Salary oncosts	9,148	8,995	8,855	9,339
C	Qualifications	-	-	-	-
D	Overheads	25,429	25,803	26,127	26,705
E	Capital overheads	5,376	5,455	4,377	4,474
F	Travel	-	-	-	-
G	Total costs (∑A–F)	71,361	72,412	72,174	73,771
Working time					
H	Number of hours worked per annum	1,689	1,679	1,683	1,684
I	Ratio of direct to indirect time	-	-	-	-
Unit costs		€	€	€	€
J	Unit cost per hour (G/H)	42	43	43	44

**3.1.4.5 Health Care Support Assistant (HCSA) (former Home Help)**

**Table 22a.** Estimated unit costs for a publicly employed, community based, Health Care Support Assistant, 2016–2019.

BASELINE (OVERHEADS 40%)		2016	2017	2018	2019
Cost component		€	€	€	€
A	Wages/salary	28,779	29,529	30,151	30,554
B	Salary oncosts	8,381	8,259	8,136	8,581
C	Qualifications	-	-	-	-
D	Overheads	11,511	11,812	12,061	12,222
E	Capital overheads	4,926	5,009	4,022	4,111
F	Travel	-	-	-	-
G	Total costs (∑A–F)	53,597	54,608	54,370	55,468
Working time					
H	Number of hours worked per annum	1,689	1,679	1,683	1,684
I	Ratio of direct to indirect time	-	-	-	-
Unit costs		€	€	€	€
J	Unit cost per hour (G/H)	32	33	32	33

Cost component		Description	Sources
A	Wages/salary	Annual mean whole-time equivalent basic salary for Health Care Support Assistant (HCSA), formerly Home Help.	HSE (2020) Health Service Personnel Census HSE, 2020a
B	Salary oncosts	Pay Related Social Insurance (PRSI) Contribution, calculated at 10.75–10.95% of annual mean WTE basic salary for HCSA for the period 2016—2019. Superannuation: weighted average of the public sector pension contribution rates for pre-2013 and post-2013 pension cohorts estimated by DPER (with adjustment for the Pension Related Deduction/Annual Superannuation Charge). Average pension contribution of 15–19% over the period 2016—2019 for publicly employed HCSAs.	DEASP, 2019, DEASP, 2020 DPER, 2017a DPER, 2017b Personal communication with IGEES, DOH, 04/01/2021 Personal communication with IGEES, DOH, 22/01/2021
C	Qualifications	Not included in this analysis	
D	Overheads	Estimated overhead costs for utilities (e.g., light, heat, telephone, internet), accommodation costs, office facilities, and general supplies as well as administrative and management staff costs using available guidelines. Baseline estimate of 40% of basic salary.	DOT, 2009 HIQA, 2018b HIQA, 2020 DPER, 2019 Curtis & Burns, 2019
E	Capital overheads	Estimated capital overhead costs based on average capital costs estimated by the Personal Social Services Research Unit. Estimate of 11–13% of total salary (basic plus oncosts) for the period 2016—2019.	Curtis & Burns, 2018; Curtis & Burns, 2019
F	Travel	Not included in this analysis	
H	Number of hours worked per annum	Total number of days worked per annum (number of working days in a given year minus annual leave entitlement and estimated sickness absence days) multiplied by number of hours worked per day.	Leave entitlement: HSE, 2009a; Personal communication with HSE, 04/02/2021 Absenteeism: HSE, 2017; HSE, 2018; HSE, 2019a HSE, 2019b Number of hours per week/day: Labour Relations Committee, 2013
I	Ratio of direct to indirect time	Not included in this analysis	

**Table 22b.** Estimated unit costs for publicly employed, community based, Health Care Support Assistant, 2016–2019, SENSITIVITY LOW OVERHEADS.

LOW (OVERHEADS 25%)		2016	2017	2018	2019
Cost component		€	€	€	€
A	Wages/salary	28,779	29,529	30,151	30,554
B	Salary oncosts	8,381	8,259	8,136	8,581
C	Qualifications	-	-	-	-
D	Overheads	7,195	7,382	7,538	7,639
E	Capital overheads	4,926	5,009	4,022	4,111
F	Travel	-	-	-	-
G	Total costs (∑A–F)	49,280	50,179	49,847	50,885
Working time					
H	Number of hours worked per annum	1,689	1,679	1,683	1,684
I	Ratio of direct to indirect time	-	-	-	-
Unit costs		€	€	€	€
J	Unit cost per hour (G/H)	29	30	30	30

**Table 22c.** Estimated unit costs for publicly employed, community based, Health Care Support Assistant, 2016—2019, SENSITIVITY HIGH OVERHEADS.

HIGH (OVERHEADS 60+%)		2016	2017	2018	2019
Cost component		€	€	€	€
A	Wages/salary	28,779	29,529	30,151	30,554
B	Salary oncosts	8,381	8,259	8,136	8,581
C	Qualifications	-	-	-	-
D	Overheads	23,299	23,693	24,006	24,538
E	Capital overheads	4,926	5,009	4,022	4,111
F	Travel	-	-	-	-
G	Total costs (∑A–F)	65,385	66,490	66,315	67,784
Working time					
H	Number of hours worked per annum	1,689	1,679	1,683	1,684
I	Ratio of direct to indirect time	-	-	-	-
Unit costs		€	€	€	€
J	Unit cost per hour (G/H)	39	40	39	40

### 3.2 Non-micro costed unit costs

***3.2.1 General Practitioner (GP) public.*** See Table 23 for estimated unit costs for public GP visits over the period 2016–2019.

**Table 23.** Estimated unit cost for public GP visit, 2016–2019.

Cost component		2016	2017	2018	2019
		€	€	€	€
A	Average payment to GPs per eligible person				
	Scenario 1 – Baseline	237.68	247.64	255.72	270.34
	Scenario 2 – Sensitivity	250.66	261.57	272.05	283.37
Working time					
B	Average number of GP visits per annum 2016—2019	5.91	5.91	5.91	5.91
Unit costs		€	€	€	€
C	Unit cost per public GP visit				
	Scenario 1 – Baseline	40	42	43	46
	Scenario 2 – Sensitivity	42	44	46	48

Cost component		Description	Sources
A	Average payment to GPs per eligible person	Total annual payments to GPs by PCRS for treatment of MC and GPVC holders divided by total number of eligible MC and GPVC holders as at 31st December in each year.	Total payments to GPs: PCRS, 2016; PCRS, 2017; PCRS, 2018; PCRS, 2019 Matenity & Infant Care Scheme: PCRS, 2019 MC & GPVC holders: https://www.sspcrs.ie/portal/annual-reporting [last accessed 08/12/2020]
B	Average number of GP visits per annum (Medical Card holders & GP Visit Card holders)	Weighted average GP utilisation per annum: Average number of GP visits per annum by MC and by GPVC holders disaggregated by age weighted by number of MC and GPVC holders in each age group	GP utilisation rates: Healthy Ireland, 2016; Healthy Ireland, 2018; Healthy Ireland, 2019 MC & GPVC holders by age group: https://www.sspcrs.ie/portal/annual-reporting [last accessed 08/12/2020]

Note: GP = General Practitioner; PCRS = Primary Care Reimbursement Service; MC = Medical Card; GPVC = GP Visit Card.

***3.2.2 GP private.*** See Table 24 for estimated unit costs for private GP visits over the period 2016–2019.

**Table 24.** Estimated unit cost for private GP visit, 2016–2019.

Cost component		2016	2017	2018	2019
		€	€	€	€
A	Average fee charged to private patients per GP visit	na	na	50.00	50.00

Note: na = not available

**Table:** 

Cost component		Description	Sources
A	Average fee charged to private patients per GP visit	Average payment made to GPs by non-medical card holders in the fifth wave of the Healthy Ireland Survey, covering the time period September 2018 to September 2019.	Healthy Ireland, 2019

Note: GP = General Practitioner

***3.2.3 Dentist public.*** See Table 25 for the schedule of fees payable to private dentists under the DTSS over the period 2016–2019 for public dental costs.

**Table 25.** Schedule of fees payable to private dentists under the Dental Treatment Services Scheme (DTSS), 2016–2019.

Cost component		2016	2017	2018	2019
		€	€	€	€
A	Examination	33.00	33.00	33.00	33.00
B	Prophylaxis	31.00	31.00	31.00	31.00
C	Exodontics (Extraction under local anaesthetic)	39.50	39.50	39.50	39.50

Cost component		Description	Sources
A	Examination	Fee paid to private dentists for oral examination for medical cardholders under the DTSS	PCRS 2016; PCRS, 2017; PCRS, 2018; PCRS, 2019
B	Prophylaxis	Fee paid to private dentists for prophylaxis (scale & polish) for medical cardholders under the DTSS	PCRS 2016; PCRS, 2017; PCRS, 2018; PCRS, 2019
C	Exodontics (Extraction under local anaesthetic)	Fee paid to private dentists for routine extractions (under local anaesthetic) for medical cardholders under the DTSS	PCRS 2016; PCRS, 2017; PCRS, 2018; PCRS, 2019

***3.2.4 Dentist private.*** See Table 26 for estimated average private dental prices for specific services in the year 2020.

**Table 26.** Average private dental prices for specified services, 2020.

Cost component		2020
		€
A	Examination with Dentist	43
B	Cleaning (scale & polish) with Dentist	63
C	Routine extraction with Dentist	100

Cost component		Description	Sources
A	Examination	Average price charged for private dental examination/consultation, excluding x-rays	Survey of private dentists in Ireland, preliminary results (personal communication with Smith and Jiang, 31/01/2021).
B	Cleaning (scale & polish)	Cleaning (scale & polish) with dentist	Survey of private dentists in Ireland, preliminary results (personal communication with Smith and Jiang, 31/01/2021).
C	Routine extraction	Routine extraction with dentist, average fee charged	Survey of private dentists in Ireland, preliminary results (personal communication with Smith and Jiang, 31/01/2021).

***3.2.5 Long-term residential care (LTRC).*** See Table 27 for estimated unit costs of LTRC in public and private facilities over the period 2016–2020.

**Table 27.** Estimated unit costs for long-term residential care, public and private, 2016–2020.

Cost component		2016	2017	2018	2019	2020
		€	€	€	€	€
A	Average public cost per week	1,407	1,500 ^a^	1,592	1,615	1,665
B	Average private charge per week	919	942	968	992	1,012

Note: ^a^estimated

**Table:** 

Cost component		Description	Sources
A	Average public cost per week	Costs of care including: pay (management, nursing and support staff) excluding superannuation costs, plus operating expenses to cover minor capital works, general equipment and furniture, training and education costs.	https://www.hse.ie/eng/services/news/media/pressrel/updated-costs-for-public-nursing-homes-announced-by-hse.html [last accessed 21/12/2020]; https://www.hse.ie/eng/services/news/media/pressrel/hse-publishes-cost-of-providing-care-in-public-residential-services-for-older-people.html [last accessed 21/12/2020]; https://data.oireachtas.ie/ie/oireachtas/committee/dail/32/committee_of_public_accounts/submissions/2017/2017-01-19_correspondence-hse-nursing-homes-ireland_en.pdf [last accessed 21/12/2020]
B	Average private charge per week	Agreed maximum price negotiated between each facility and the National Treatment Purchase Fund (NTPF).	https://www2.hse.ie/file-library/fair-deal/cost-of-voluntary-and-private-nursing-homes.pdf [last accessed 04/11/2020]; personal communication with NTPF, 05/11/2020; C&AG, 2020; NTPF, 2017

## 4 Conclusions & next steps

### 4.1 Concluding comments

This paper presents detailed unit costs for 16 healthcare professionals in community-based non-acute services in Ireland. A central national database of unit costs for non-acute healthcare has not previously been available for Ireland. The unit costs set out in this paper can be used in a wide range of health costing studies in the Irish context (e.g., economic evaluations, cost-of-illness studies). The availability of a set of transparent costs will ensure consistency across Irish health costing studies and facilitate cross-study and cross-country comparisons.

### 4.2 Limitations

The unit costs included here are based on the best available data for 2016–2019 ^32^. There are important limitations in currently available data that need to be addressed in future analyses of unit costs and these have been highlighted where relevant throughout the paper.

First, for the micro-costed unit costs, there remain data gaps around some of the core cost components that have been included in this paper. In particular, there are no published detailed data on overhead costs in community-based healthcare facilities in Ireland. While the paper has presented alternative overhead rates, the baseline of 40% provides a reasonable benchmark between the high rates applied in the UK and the comparatively low rate recommended in current Irish costing guidelines.

Second, there is need to advance data collection for some key services (e.g., additional nursing cadres working in the community) and cost components that have not been included in this paper, in particular, qualification costs, and travel costs.

Third, there is need to advance analysis around the core salary cost component. Assessment of total earnings rather than basic salary together with analysis of actual rather than contracted working hours would give important insight into the extent of overtime that staff are working. The cost of agency staff is also not considered in this paper and is an important consideration in the context of considerable reliance on agency staff in the Irish healthcare workforce ( Houses of the Oireachtas Committee on the Future of Healthcare, 2017).

Fourth, this paper has focused on providing a unit cost per hour of service while in practice there is variability in duration of visits both within and across services. For example, an initial physiotherapy assessment might last 1 hour while subsequent visits might last 30 minutes ( Brick *et al.*, 2010), or a GP visit can last a few minutes or much longer depending on the complexity of the presenting complaint ( Pierse *et al.*, 2019). As discussed earlier, the increased role for GPs in chronic disease prevention, diagnosis and management as part of the new 2019 GP Agreement ( DOH *et al.*, 2019) may have implications for the duration of GP visits.

Thus, as noted at the outset, the set of costs included in this paper should be seen as a fluid set of data where improvements need to be made over time. In the meantime, transparency in methods and data sources ensures that users of these unit costs are better able to make cross-study and cross-country comparisons.

### 4.3 Next steps

This paper brings Ireland in line with other countries that already have a central database of unit costs for non-acute care (e.g., UK) and will facilitate future engagement with international efforts to standardise cost-calculation methods across Europe.

The unit costs for the publicly employed non-acute healthcare professionals presented in this paper have changed by 2–6% over the timeframe 2016–2019. Larger percentage changes are observed in the unit costs for public GP visits and public LTRC (14–15%) over the period 2016–2019. As discussed, future patterns in GP costs are difficult to project because of the implications of the new 2019 GP Agreement ( DOH *et al.*, 2019) and further examination of the public LTRC costs will be required in light of the outcomes of the LTRC value-for-money review. While the patterns observed in the unit costs presented in this paper provide reasonable estimates of the likely costs for these services for 2020–2021, it is important to recognise that for these cost data to make useful contributions to decision-making in healthcare research, they need to be updated on a regular basis with specific need for more in-depth analysis of changes to GP and LTRC costing structures. Moreover, the implications of the COVID-19 pandemic for the costs of delivering community-based non-acute healthcare (e.g., increased phone and online consultations) will need to be assessed.

The analysis also suggests that current Irish health costing guidelines need to be revised, particularly in terms of pension costs.

As outlined above, there is need for more detailed data and analysis in the following areas relating to the costs of delivering community-based non-acute care:

-   Overhead costs;

-   Qualification costs for community-based non-acute healthcare professionals;

-   Travel costs;

-   Additional salary costs (over and above basic salary) associated with overtime, agency staff, etc.;

-   Duration of consultations (average duration for each service as well as examination of sources of variation in durations).

The joint tasks of addressing these data gaps as well as updating the unit costs will require dedicated resources for the development of an ongoing database of unit costs for non-acute care. Linking in with the research by the PECUNIA project will also ensure consistency in methods and comparability in costs in cross-country studies within Europe.

## Data availability statement

### Source data

The analysis in this paper is based on secondary data sources that are available from the DOH, the HSE, other public administrative bodies, and publicly available survey data.

- For the micro-costed unit costs, the primary data points include basic salaries for selected healthcare professionals, at selected grades, and these are drawn from the HSE consolidated salary scales and informed by data provided by the HSE Personnel Census. Superannuation estimates are generated using data on the proportion of HSE staff on pre- and post-2013 pension schemes. Data on the number of active HSE (including Section 38) Single Scheme members at December of each year are deducted from the total number of HSE (including Section 38) employees to estimate the proportion of employees in the pre- and post-2013 pension cohorts for the years 2016–2019. Data on Single Scheme membership were provided by the DOH and DPER. HSE headcount data for the years 2016–2019 were also provided by the DOH. Annual leave entitlements are identified from the HSE as well as from national agreements. Absenteeism rates for HSE staff working in community services, disaggregated by staff category, are available in HSE Management Data Reports.
- For GP costs, data on the number of MC and GPVC holders are available from the PCRS (online portal and in published annual reports). Data on GP utilisation are available from the HI surveys for the years 2016, 2018 and 2019. Data on private payments to GPs are available in the fifth wave of the HI survey (2018–2019).
- For dental care, the schedule of fees payable to dentists under the DTSS is publicly available in the PCRS annual reports. Preliminary findings from a 2020 survey of private dental fees by the authors of this paper are also included and the final results from the survey are due to be made publicly available this year.
- For LTRC, charges for public LTRC facilities are published by the HSE and the charges for private LTRC facilities are published by the NTPF.

## Footnotes

^1^ With the exceptions of LTRC and private dental care, for which unit costs for 2020 are also provided.

^2^ http://www.hpo.ie/seminar/pdf/2019/ABF_Conf_2019_Brian_Donovan.pdf [last accessed 08/10/2020]

^3^ ProgrammE in Costing, resource use measurement and outcome valuation for Use in multi-sectoral National and International health economic evaluAtions https://www.pecunia-project.eu/ [last accessed 21/12/2020].

^4^ https://www.gov.ie/en/publication/public-spending-code/ [last accessed 02/12/2020]

^5^ Alternative methods are used for more complex services (e.g., care packages, residential care homes, etc.).

^6^ Prior to 2013, the PSSRU used total earnings for hospital-based doctors (divided by hours worked including overtime) to reflect the high proportion of doctors working long hours relative to other staff cadres ( Curtis, 2013), but this changed in 2013 following a change in the source data.

^7^ https://www.hse.ie/eng/staff/benefitsservices/pay/increments.html [last accessed 19/11/2020]

^8^ Note that this aligns with the HIQA guidelines (for budget impact and economic evaluations of health technologies) which suggest a pension rate of 4% (of basic salary) although there is no mention of the Single Public Service Pension Scheme in the guidelines ( HIQA, 2018b; HIQA, 2020).

^9^ Based on a pensionable salary of €50,000 the total annual ASC payment would be €1,800 (equivalent to 4% of total salary) for an employee with a standard accrual pension; and €1,199 (equivalent to 2% of total salary) for an employee with a Single Public Service Pension.

^10^ Personal communication with Irish Government Economic and Evaluation Service (IGEES), DOH, 04/01/2021.

^11^ Personal communication with IGEES, DOH, 22/01/2021.

^12^ Personal communication with IGEES, DOH, 04/01/2021.

^13^ Although not documented, the source for this estimate is understood to be a 2003 report on research overheads by Forfás and the Higher Education Authority (Personal communication with IGEES, DOH, 29/10/2020). The report recommended an overhead rate of 25% for desk-based research, and 30% for laboratory-based research ( HEA & Forfás, 2003).

^14^ Note that annual absenteeism rates by staff category for community services are available in the January HSE Management Data Reports ( HSE, 2017; HSE, 2018; HSE, 2019a). These rates include HSE as well as ‘Section 38’ staff (a range of non-acute and voluntary hospitals provided with HSE funding under Section 38 of the Health Act 2004 ( HSE, 2020b). In contrast, the WTE data in the HSE Personnel Census that are used to select the grade code with the largest number of WTEs include HSE staff only.

^15^ The HIQA guidelines ( HIQA, 2018b; HIQA, 2020) refer to Department of Taoiseach RIA 2009 guidelines ( DOT, 2009) for the calculation of hours worked per week, while the DPER 2019 guidelines refer to more recent data on working hours ( DPER, 2013) in which the majority of civil servants had their working week increased to 37 hours per week (or 7.4 hours per day) following the 2013 Public Service Stability Agreement ( Labour Relations Committee, 2013). Also note that the 2013 Agreement refers to the increase in the working week for nurses and midwives to 39 hours per week, or 7.8 hours per day ( Labour Relations Committee, 2013).

^16^ The 1989 contract for MC holders and the related 2005 contract for GPVC holders are together referred to as the GMS contracts and the contract for children under 6 is referred to as the Under 6s contract ( DOH *et al.,* 2019). There are plans, as part of the Sláintecare Reform programme, to undertake a fundamental review of the contractual framework for GP services to ensure a sustainable GP service as a core element of primary care and one that facilitates integrated healthcare delivery ( DOH *et al.,* 2019).

^17^ MC where *i*=0, GPVC (including Under 6s) where *i*=1

^18^ 0–5, 6–11, 12–15, 16–44, 45–64, 65+

^19^ https://www.sspcrs.ie/portal/annual-reporting/report/eligibility [last accessed 02/09/2020]

^20^ https://www.sspcrs.ie/portal/annual-reporting/report/eligibility [last accessed 02/09/2020]

^21^ Personal communication with DOH and Ipsos MRBI, 17/12/2020

^22^ Personal communication with Smith and Jiang, 31/01/2021.

^23^ All LTRC facilities are required to register with HIQA ( C&AG, 2020).

^24^ https://www.hse.ie/eng/services/news/media/pressrel/updated-costs-for-public-nursing-homes-announced-by-hse.html [last accessed 21/12/2020]; https://www.hse.ie/eng/services/news/media/pressrel/hse-publishes-cost-of-providing-care-in-public-residential-services-for-older-people.html [last accessed 21/12/2020]; https://data.oireachtas.ie/ie/oireachtas/committee/dail/32/committee_of_public_accounts/submissions/2017/2017-01-19_correspondence-hse-nursing-homes-ireland_en.pdf [last accessed 21/12/2020]

^25^ https://www2.hse.ie/file-library/fair-deal/cost-of-voluntary-and-private-nursing-homes.pdf [last accessed 04/11/2020]; personal communication with NTPF, 05/11/2020; C&AG, 2020; NTPF, 2017.

^26^ Progress with the value-for-money review has been delayed by concerns over data protection. https://www.irishtimes.com/news/health/nursing-home-fair-deal-review-paused-over-data-protection-concerns-1.4369575 [last accessed 29/10/2020]).

^27^ Including HSE and Section 38 services (a range of non-acute and voluntary hospitals provided with HSE funding under Section 38 of the Health Act 2004 ( HSE, 2020b).

^28^ Source: Health Service Personnel Census, HSE

^29^ Further analysis of data from the Health Service Personnel Census is required before the unit costs for Speech & Language Therapists and for Social Workers can be calculated.

^30^ Including HSE and Section 38 services.

^31^ Further analysis of data from the Health Service Personnel Census is required before the unit costs for other nursing cadres can be calculated.

^32^ With the exceptions of LTRC and private dental care, for which unit costs for 2020 are also provided.
