# Supplementary material for: Unit costs for non-acute care in Ireland 2016—2019
Source: HRB Open Res. 2021 Apr 23;4:39. [Version 1] doi: 10.12688/hrbopenres.13256.1 (PMC8917322; doi:10.12688/hrbopenres.13256.1)
Supplement: Peer review history

# Peer review: Reviewer response for version 1

**DOI:** 10.21956/hrbopenres.14422.r31412

**Published:** 2022-03-11

**Authors:** Aileen Murphy

**Affiliations:** 1 Department of Economics, Cork University Business School, University College Cork, Cork, Ireland

- This work provides a pragmatic and useful estimation of unit costs in Ireland - a long awaited resource. The authors should be commended.
- A very good overview of costing methodologies was presented at the outset. 
- Authors indicate future iterations will consider practice nurses as standalone - this will be very important going forward as care shifts to the community and primary care services face increasing demands.
- Given issues surrounding the availability of GP utilisation amongst minors, authors could consider if the Growing up in Ireland (GUI) dataset provides any insights. 
- Finally, given the useful nature of the data provided here have the authors considered presenting the data on a dashboard/website which could be updated periodically and cited to increase circulation and accessibility? 

Is the work clearly and accurately presented and does it cite the current literature?

Yes

If applicable, is the statistical analysis and its interpretation appropriate?

Yes

Are all the source data underlying the results available to ensure full reproducibility?

Yes

Is the study design appropriate and is the work technically sound?

Yes

Are the conclusions drawn adequately supported by the results?

Yes

Are sufficient details of methods and analysis provided to allow replication by others?

Yes

Reviewer Expertise:

Health economics

I confirm that I have read this submission and believe that I have an appropriate level of expertise to confirm that it is of an acceptable scientific standard.

# Peer review: Reviewer response for version 1

**DOI:** 10.21956/hrbopenres.14422.r29328

**Published:** 2021-07-16

**Authors:** Conor Teljeur

**Affiliations:** 1 Health Technology Assessment (HTA) Directorate, Health Information and Quality Authority, Dublin, Ireland

This is an important piece of work that provides unit cost data on a range of non-acute healthcare professionals in Ireland. The paper has clear aims, is clearly written and well referenced. I have some comments or queries on specific sections.

1. Superannuation.
  The paper includes superannuation for HSE-employed individuals. The standard has been a low rate of contribution in the region of 4%. More recent DPER guidance suggests that the true cost may be substantially higher. The actuarial approach adopted was applied to only two healthcare grades and the calculations were applied to a generic case. Future work or research needs to look at the applicability of their approach and findings to a range of healthcare professionals and grades.
2. GP utilisation data.
  It would be helpful to state what the recall period is for the GP utilisation data. Longer recall periods are subject to greater bias. It would also be useful to know if there is potential for seasonal bias, for example, if the survey is predominantly conducted during the winter months. I understand it is conducted over most of the year but it would be useful to clarify.
3. Private GP cost.
  HIQA have previously used data from www.WhatClinic.com which included cost data for a subset of 644 practices. The data were not validated and it is apparent that there may not be something that can be defined as a standard visit. Within private practice there are also approaches to managing visit costs, such as a reduced "repeat visit" fee, or delegation of certain duties to a practice nurse. While this makes estimating a true cost challenging, it has to come back to what the cost is being used for. If it is to understand what an individual will have to pay out of pocket to access a GP consultation, the standard visit cost is probably most appropriate.

Again, I must commend the authors for the work they have put into this, and is likely to become a well used and cited paper.

Is the work clearly and accurately presented and does it cite the current literature?

Yes

If applicable, is the statistical analysis and its interpretation appropriate?

Yes

Are all the source data underlying the results available to ensure full reproducibility?

Yes

Is the study design appropriate and is the work technically sound?

Yes

Are the conclusions drawn adequately supported by the results?

Yes

Are sufficient details of methods and analysis provided to allow replication by others?

Yes

Reviewer Expertise:

Health economics; health services research; health inequalities; primary care; public health

I confirm that I have read this submission and believe that I have an appropriate level of expertise to confirm that it is of an acceptable scientific standard.
